# Supplementary material for: Valine aminoacyl-tRNA synthetase promotes therapy resistance in melanoma
Source: Nat Cell Biol. 2024 Jun 7;26(7):1154–64. doi: 10.1038/s41556-024-01439-2 (PMC11252002; doi:10.1038/s41556-024-01439-2)
Supplement: Supplementary file 1 — Supplementary Information 1. [file 41556_2024_1439_MOESM1_ESM.pdf]

nature cell biology

Supplementary information

https://doi.org/10.1038/s41556-024-01439-2

Valine aminoacyl-tRNA synthetase promotes therapy resistance in melanoma

In the format provided by the authors and unedited

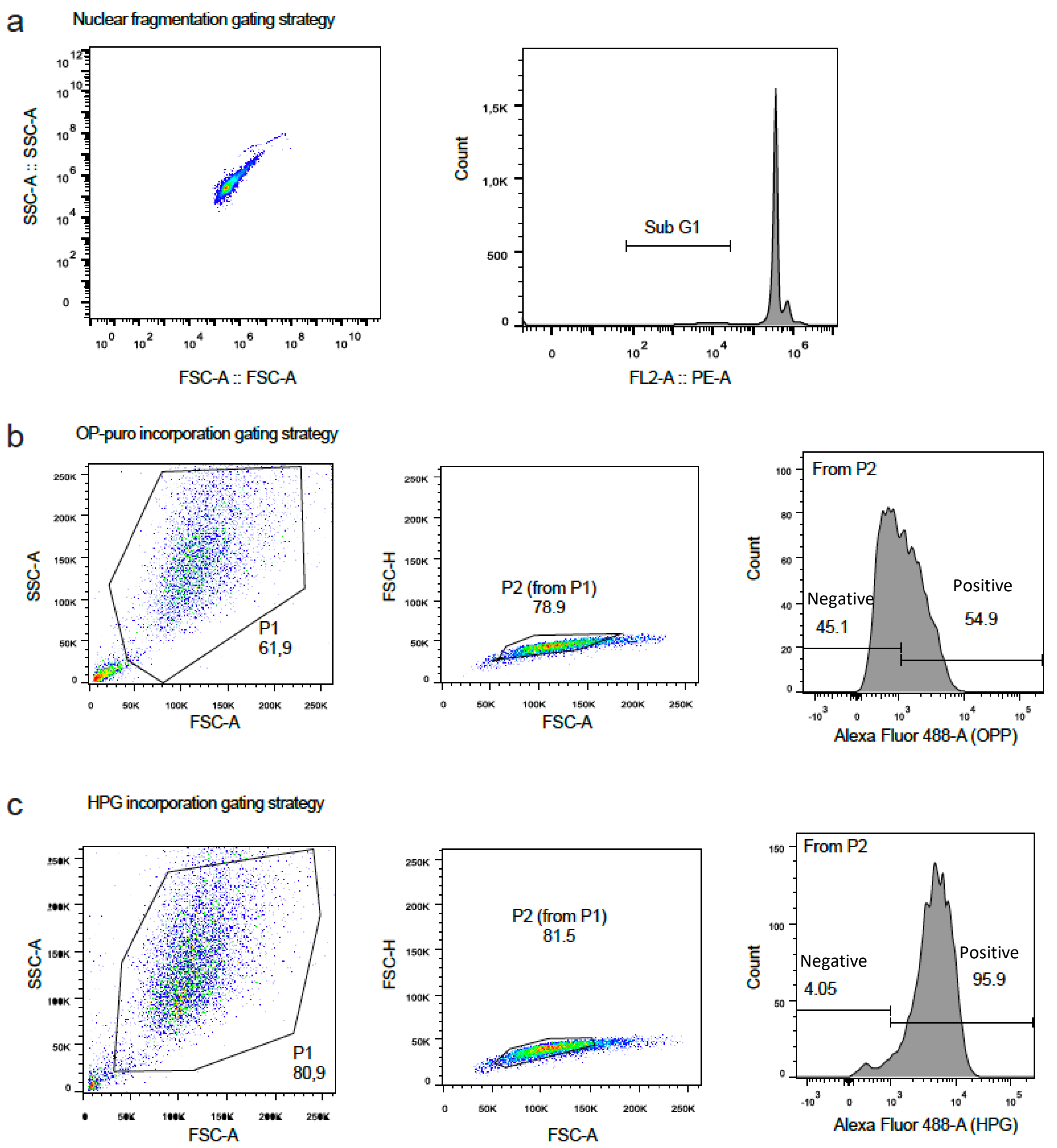

Supplementary information

Supplementary information: Gating strategy for FACS experiments. a, DNA fragmentation of cells stained with PI assessed as shown. No previous gating was performed (Sub G1 corresponds to dead cells). **b,** OP-puro (OPP) incorporation assay and **c,** HPG incorporation assay whereas P1 represents all cells without debris. P2 (from P1) represents single cells. The last gating represents the percentage of positive and negative cells for OPP or HPG respectively.
