# Supplementary material for: Valine aminoacyl-tRNA synthetase promotes therapy resistance in melanoma
Source: Nat Cell Biol. 2024 Jun 7;26(7):1154–64. doi: 10.1038/s41556-024-01439-2 (PMC11252002; doi:10.1038/s41556-024-01439-2)
Supplement: Supplementary file 2 — Reporting Summary [file 41556_2024_1439_MOESM2_ESM.pdf]

nature portfolio

Corresponding author(s): Pierre CLOSE

Last updated by author(s): May 11, 2024

Reporting Summary

Nature Portfolio wishes to improve the reproducibility of the work that we publish. This form provides structure for consistency and transparency in reporting. For further information on Nature Portfolio policies, see our Editorial Policies and the Editorial Policy Checklist.

Statistics

For all statistical analyses, confirm that the following items are present in the figure legend, table legend, main text, or Methods section.

n/a	Confirmed	
☐	☒	The exact sample size (*n*) for each experimental group/condition, given as a discrete number and unit of measurement
☐	☒	A statement on whether measurements were taken from distinct samples or whether the same sample was measured repeatedly
☐	☒	The statistical test(s) used AND whether they are one- or two-sided<br>*Only common tests should be described solely by name; describe more complex techniques in the Methods section.*
☐	☒	A description of all covariates tested
☐	☒	A description of any assumptions or corrections, such as tests of normality and adjustment for multiple comparisons
☐	☒	A full description of the statistical parameters including central tendency (e.g. means) or other basic estimates (e.g. regression coefficient) AND variation (e.g. standard deviation) or associated estimates of uncertainty (e.g. confidence intervals)
☐	☒	For null hypothesis testing, the test statistic (e.g. *F*, *t*, *r*) with confidence intervals, effect sizes, degrees of freedom and *P* value noted<br>*Give P values as exact values whenever suitable.*
☒	☐	For Bayesian analysis, information on the choice of priors and Markov chain Monte Carlo settings
☒	☐	For hierarchical and complex designs, identification of the appropriate level for tests and full reporting of outcomes
☒	☐	Estimates of effect sizes (e.g. Cohen's *d*, Pearson's *r*), indicating how they were calculated

Our web collection on statistics for biologists contains articles on many of the points above.

Software and code

Policy information about availability of computer code

Data collection	- Homo sapiens transcriptome was downloaded from the human genome database (HGD-ENSEMBL) for codon and amino acid enrichment analysis.
Data analysis	- All statistical tests: chi-square test, student's t test, ANOVA test were performed on Excel 2016 or GraphPad Prism 10.<br>- Codon enrichment was evaluated using a chi-squared test, comparing the codon distribution of each of the genes from each group to the human transcriptome. The same was applied for the amino acid enrichment analysis.<br>- Gene enrichment pathways were assessed by using Gene Set Enrichment Analysis (GSEA). Bubble plots representing the pathways were generated using R or GraphPad Prism.<br>- GEPIA2 tool was used to assess survival analysis and expression of aaRSs (in The Cancer Genome Atlas (TCGA) database).<br>- For the establishment of VARS signature: A custom R script using single-sample GSEA (ssGSEA) and a differential expression analysis with Limma R package's (v3.56.1) was used in this study.<br>- For proteomics analysis the following software were used: MaxQuant software v1.6.0.16<br>- QuPath 0.3.2 is used for the quantification of the immunolabelled melanoma cells.<br>- Diricore analysis for codon occupancy was used from github (https://github.com/pkorner218/Ribosome_Diricore_pipeline/).

For manuscripts utilizing custom algorithms or software that are central to the research but not yet described in published literature, software must be made available to editors and reviewers. We strongly encourage code deposition in a community repository (e.g. GitHub). See the Nature Portfolio guidelines for submitting code & software for further information.

Data

Policy information about availability of data

All manuscripts must include a data availability statement. This statement should provide the following information, where applicable:

- Accession codes, unique identifiers, or web links for publicly available datasets
- A description of any restrictions on data availability
- For clinical datasets or third party data, please ensure that the statement adheres to our policy

Polysome and RNA sequencing: Gene Expression Omnibus; accession number GSE236046;
Ribosome sequencing: Gene Expression Omnibus; accession number GSE236642;
tRNA sequencing: Gene Expression Omnibus; accession number GSE236645;

Proteomics data: The Mmass spectrometry data have been deposited in ProteomeXchange with the primary accession code PXD044863 (https://www.ebi.ac.uk/pride/archive/projects/PXD044863/private) and PXD044910 (https://www.ebi.ac.uk/pride/archive/projects/PXD044910/private).

For the establishment of VARS signature: A custom R script using single-sample GSEA (ssGSEA) and a differential expression analysis with Limma R package's (v3.56.1) was used in this study.

Research involving human participants, their data, or biological material

Policy information about studies with human participants or human data. See also policy information about sex, gender (identity/presentation), and sexual orientation and race, ethnicity and racism.

Reporting on sex and gender	8 biopsies from women and 7 biopsies from men were available. Sex and gender were not considered in the study design nor in the data analysis.
Reporting on race, ethnicity, or other socially relevant groupings	N/A
Population characteristics	Human biopsy samples from male and female patients of age range between 26 and 81 years old were retrieved from the biobank of the University Hospital Center in Liege. Patients were all diagnosed with melanoma, and were tested positive for BRAF mutation. All samples used were obtained from leftover biopsy samples when available and did not interfere with standard practices of care (12 samples were obtained for normal skin, 12 samples for primary melanoma and 21 samples for metastatic melanoma).
Recruitment	No active recruitment was performed. Leftover biopsy samples were used when available. Informed consent was obtained from the patients providing samples. The participants were not compensated.
Ethics oversight	The protocol was approved by the ethical committee of the University of Liege (#3006695).

Note that full information on the approval of the study protocol must also be provided in the manuscript.

Field-specific reporting

Please select the one below that is the best fit for your research. If you are not sure, read the appropriate sections before making your selection.

☒ Life sciences ☐ Behavioural & social sciences ☐ Ecological, evolutionary & environmental sciences

For a reference copy of the document with all sections, see nature.com/documents/nr-reporting-summary-flat.pdf

Life sciences study design

All studies must disclose on these points even when the disclosure is negative.

Sample size	Animal sample size experiment was assessed by Web Power. For the other experiments, no sample size calculation was performed.
Data exclusions	Concerning mice experiment, we performed ROUT test on GraphPad Prism to identify outliers. One mouse harbouring shCTRL tumor on one flank and shVARS-1 tumor on the other flank was identified as outlier and excluded from the analysis.
Replication	All experiments were performed with at least 2 biological replicates. The exact number of replicates is stated in figure legends
Randomization	Randomization was not performed, experiments were done in cell lines or xenografts were randomization is not applicable. Allocation of samples into experimental groups is not relevant, as the study helps us identify new features to discrimnate between already established experimental groups.
Blinding	Xenograft experiments were not performed in blind as these experiments are conducted for explorative purposes.

Reporting for specific materials, systems and methods

We require information from authors about some types of materials, experimental systems and methods used in many studies. Here, indicate whether each material, system or method listed is relevant to your study. If you are not sure if a list item applies to your research, read the appropriate section before selecting a response.

Materials & experimental systems

n/a	Involved in the study
☐	☒ Antibodies
☐	☒ Eukaryotic cell lines
☒	☐ Palaeontology and archaeology
☐	☒ Animals and other organisms
☒	☐ Clinical data
☒	☐ Dual use research of concern
☒	☐ Plants

Methods

n/a	Involved in the study
☒	☐ ChIP-seq
☐	☒ Flow cytometry
☒	☐ MRI-based neuroimaging

Antibodies

Antibodies used

All antibodies and dilutions used in this study are listed in supplementary table 1.
Name Antibody Host Reactivity Company Catalogue number Size (kDa) Application
VARS ValRS (D-7) Mouse H, M, Rats Santa-Cruz sc-166674 130 WB
NBP2-20843 VARS Rabbit H Novus Biological NBP2-20843 IHC
HADH HADH Rabbit H, M, Rats Invitrogen/ Thermofisher PA5-31157 34 WB/ IHC
CPT1a CPT1A (D3B3) Rabbit H Cell Signaling 12252 88 WB
GAPDH GAPDH (D16H11) Rabbit H, M, R, Mk Cell Signaling 5174 37 WB
HSP90 Hsp90 alpha/beta (H-114) Rabbit H Santa-Cruz sc-7947 90 WB
ATF4 ATF4 Rabbit H, M, R Cell Signaling 11815s 49 WB
p-EIF2alpha (Ser51) Phospho-eIF2α (Ser51) Rabbit H,M, R Cell Signaling 9721S 38 WB
EIF2alpha eIF2α Rabbit H,M, R Cell Signaling 9722 38 WB
SLC7A5 LAT1 Rabbit H Cell Signaling 5347S 39 WB
KIF13B KIF13B Rabbit H Bio-techne NBP1-83398 200 WB
QDPR QDPR (B-1) Mouse H Santa-Cruz sc-376218 26 WB
GOLT1B GOLT1B Rabbit H, M, R Gentaur DF9071 15 WB
BRAF (F-3), BRAF, Mouse, H,M,Rats, Santa cruz, sc-55522, 95, WB
MEK1/2 (L38C12), MEK 1/2, Mouse, H, M, R, Mk, Cell Signaling, 4694, 45, WB
Phospho-MEK1/2 (Ser217/221) (41G9), Phospho-MEK1/2, Rabbit, H, M, R, Mk, Cell Signaling, 9154, 45, WB
p44/p42 MAPK (Erk1/2), ERK, Rabbit, H, M, R, Hm, Mk, Mi, Z, B, Pg, Sc, Cell Signaling, 9102, 42-44, WB/IP
Phospho-p44/42 MAPK (Erk1/2) (Thr202/Tyr204), Phospho-ERK, Rabbit, H, M, R, Hm, Mk, Mi, Z, B, Pg, Sc, , Cell Signaling, 4370, 42-44, WB/ IP/ IHC/ IF/ F

Validation

Validation of the listed antibodies was performed by the manufacturer. Data is available at the manufacturer's website as indicated below:
ValRS sc-166674: https://www.scbt.com/fr/p/valrs-antibody-d-7
VARS (NBP2-20843): https://www.novusbio.com/products/vars-antibody_nbp2-20843
HADH (PA5-31157): https://www.thermofisher.com/antibody/product/HADH-Antibody-Polyclonal/PA5-31157
CPT1a (12252): https://www.cellsignal.com/products/primary-antibodies/cpt1a-d3b3-rabbit-mab/12252
GAPDH (5174): https://www.cellsignal.com/products/primary-antibodies/gapdh-d16h11-xp-rabbit-mab/5174
HSP90 (sc-7947): https://www.scbt.com/p/hsp-90alpha-antibody-f-2?gad_source=1&gclid=EAIaIQobChMIopaDxem5hQMVnLVoCR1Z_QqREAAYASAAEgJgWPD_BwE
ATF4 (11815s): https://www.cellsignal.com/datasheet.jsp?productId=11815&images=1
p-EIF2alpha (Ser51) (9721S): https://www.cellsignal.com/products/primary-antibodies/phospho-eif2a-ser51-antibody/9721
EIF2alpha (9722): https://www.cellsignal.com/products/primary-antibodies/eif2a-antibody/9722
SLC7A5 (5347S): https://www.cellsignal.com/products/primary-antibodies/lat1-antibody/5347
KIF13B (NBP1-83398): https://www.bio-techne.com/p/antibodies/kif13b-antibody_nbp1-83398
QDPR (sc-376218): https://www.scbt.com/fr/p/qdpr-antibody-b-1
BRAF (F-3) (sc-55522): https://www.scbt.com/p/raf-b-antibody-f-3?gad_source=1&gclid=EAIaIQobChMImeTrrOu5hQMVqS0GAB1hwgDzEAAYASAAEgLaj_D_BwE
MEK1/2 (L38C12) (4694): https://www.cellsignal.com/products/primary-antibodies/mek1-2-l38c12-mouse-mab/4694
Phospho-MEK1/2 (Ser217/221) (41G9) (9154): https://www.cellsignal.com/products/primary-antibodies/phospho-mek1-2-ser217-221-41g9-rabbit-mab/9154
p44/p42 MAPK (Erk1/2) (9102): https://www.cellsignal.com/products/primary-antibodies/p44-42-mapk-erk1-2-antibody/9102
Phospho-p44/42 MAPK (Erk1/2) (Thr202/Tyr204) (4370): https://www.cellsignal.com/products/primary-antibodies/phospho-p44-42-mapk-erk1-2-thr202-tyr204-d13-14-4e-xp-174-rabbit-mab/4370

For GOLT1B antibody validation was performed in Liu et al. Cancer Cell Int (2021).

Eukaryotic cell lines

Policy information about cell lines and Sex and Gender in Research

Cell line source(s)	Cell lines source is stated in the material and methods section of the manuscript (Cell culture).<br>A375 melanoma cell lines were purchased from ATCC.<br>A375 vemurafenib-resistant cells were generated by increasing doses of vemurafenib up to 1 µM.<br>M395 (SENS and 1 µM vemurafenib RES) lines were from the laboratory of R. Lo (UCLA Division of Dermatology) - non commercial.<br>MM029, MM099 and MM383 naïve and their resistant counterparts (0.2 µM dabrafenib and 40 nM of trametinib) were provided by JC. Marine (KU Leuven) - non commercial<br>MM074 was provided by G. Ghanem (Institut J. Bordet, Université Libre de Bruxelles) - non commercial<br>Lenti-X293T cells were purchased from sigma-aldrich
Authentication	None of the cell lines used were authenticated.
Mycoplasma contamination	Mycoplasma test was performed routinely. Only negative lines are used in the study.
Commonly misidentified lines<br>(See ICLAC register)	None of the used cells are in the misidentified lines.

Animals and other research organisms

Policy information about studies involving animals; ARRIVE guidelines recommended for reporting animal research, and Sex and Gender in Research

Laboratory animals	Eight-weeks-old NOD/SCID mice. Experiments were approved by the Ethical committee of the University of Liege (#2126). The temperature and relative humidity were 21°C and 45–60%, respectively. Cages were ventilated, softly lit, and subjected to a light dark cycle.
Wild animals	no wild animal was used in thids study
Reporting on sex	Sex was not considered in the study design.
Field-collected samples	no field collected samples were used in the study.
Ethics oversight	Experiments were approved by the Ethical committee of the University of Liege (#2126).

Note that full information on the approval of the study protocol must also be provided in the manuscript.

Flow Cytometry

Plots

Confirm that:

- [x] The axis labels state the marker and fluorochrome used (e.g. CD4-FITC).
- [x] The axis scales are clearly visible. Include numbers along axes only for bottom left plot of group (a 'group' is an analysis of identical markers).
- [x] All plots are contour plots with outliers or pseudocolor plots.
- [x] A numerical value for number of cells or percentage (with statistics) is provided.

Methodology

Sample preparation	For the nuclear fragmentation assay: Supernatant and trypsinized cells were collected and stained with Nicoletti Buffer (0,1% Trinatriumcitrat-Dihydrat pH7.4, 0,1% Triton-X 100, 0.01% Propidium iodide).<br>For OPP and HPG assay: Cells were incubated with OPP or HPG (following an incubation in methionine-free media for HPG analysis) at indicated times in the material and methods section. Cells were then washed, fixed and permeabilized and the Click-iT Cell Reaction buffer Kit was used following the manufacturer's instructions.
Instrument	FACS Canto II (OPP and HPG), Cytoflex Cytometer (Nuclear fragmentation assay)
Software	FlowJo, BD FACSDiva software.
Cell population abundance	No sorting was performed

Gating strategy	The gating strategy for cell death assay, OPP and HPG are provided in supplementary information.<br>Cell death was determined as sub G1 population.<br>For HPG and OPP healthy cells were gated (FSC_SSC plot) and doublets were removed (FSC-A_FSC-H plot). Cells positive for OPP or HPG are represented in the gating strategy.

☒ Tick this box to confirm that a figure exemplifying the gating strategy is provided in the Supplementary Information.
