# Supplementary figures and images for: Valine aminoacyl-tRNA synthetase promotes therapy resistance in melanoma
Source: Nat Cell Biol. 2024 Jun 7;26(7):1154–64. doi: 10.1038/s41556-024-01439-2 (PMC11252002; doi:10.1038/s41556-024-01439-2)

Related to figure 2f

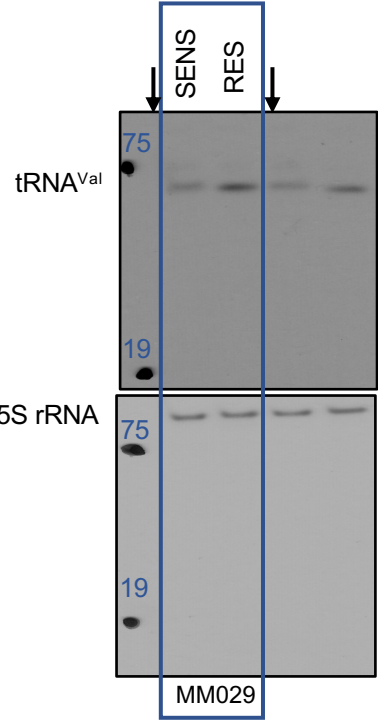

Supplement: Supplementary file 5 — Unprocessed western blots/gels. [file 41556_2024_1439_MOESM5_ESM.pdf]

Related to figure 3b

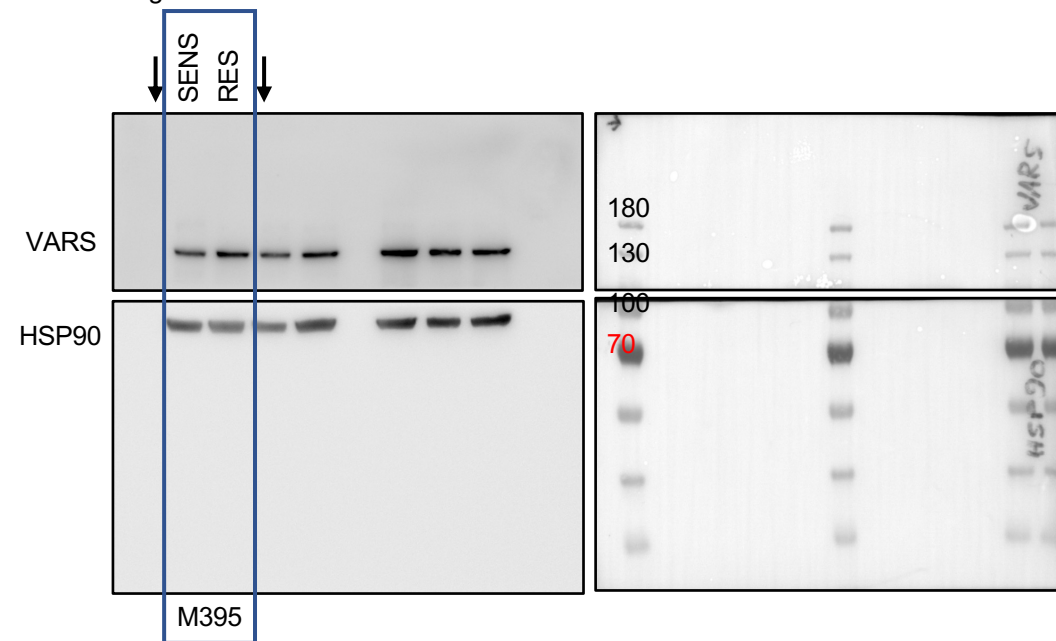

Related to figure 3d

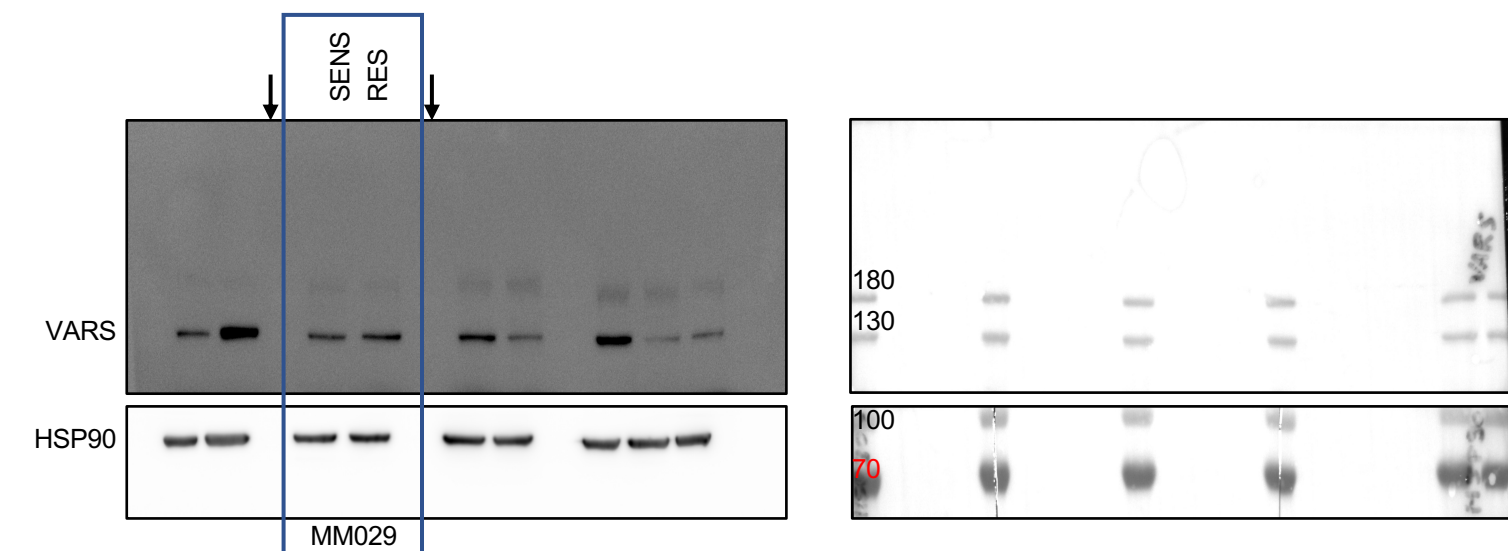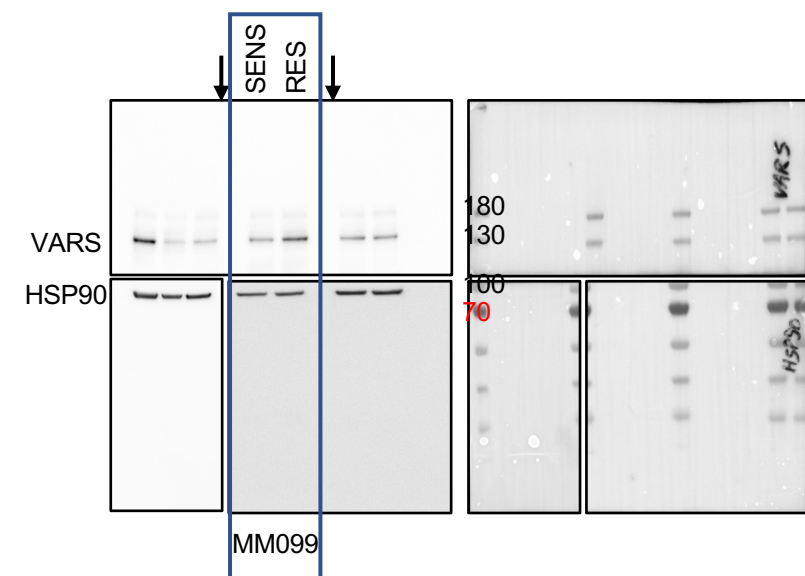

Supplement: Supplementary file 6 — Unprocessed western blots/gels. [file 41556_2024_1439_MOESM6_ESM.pdf]

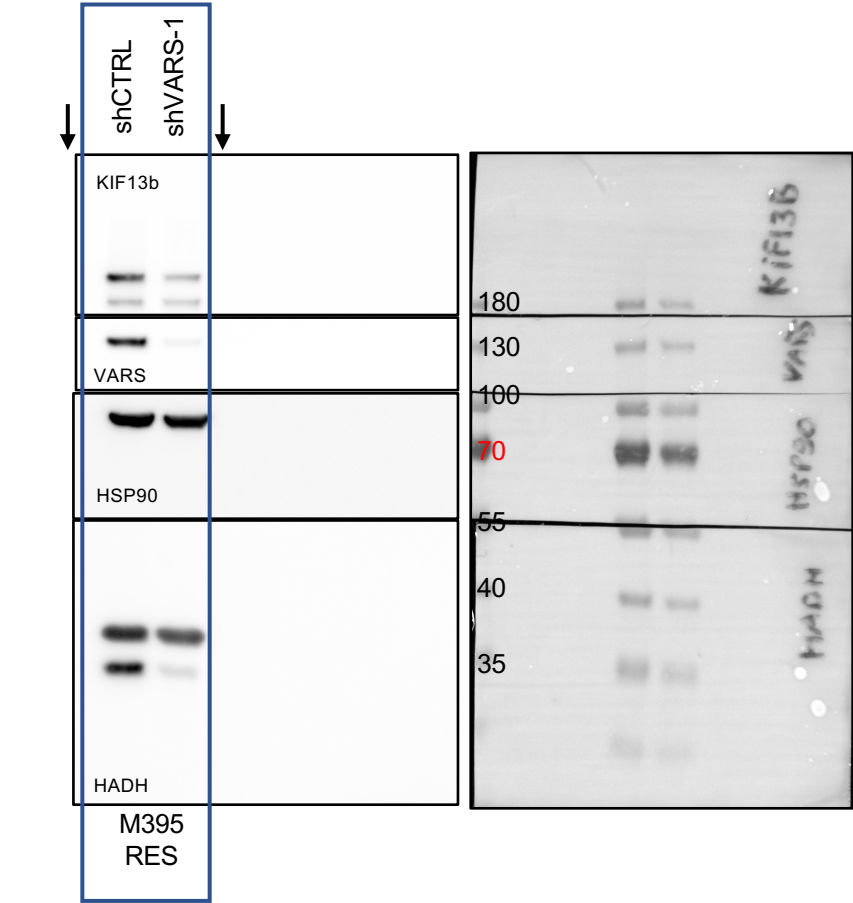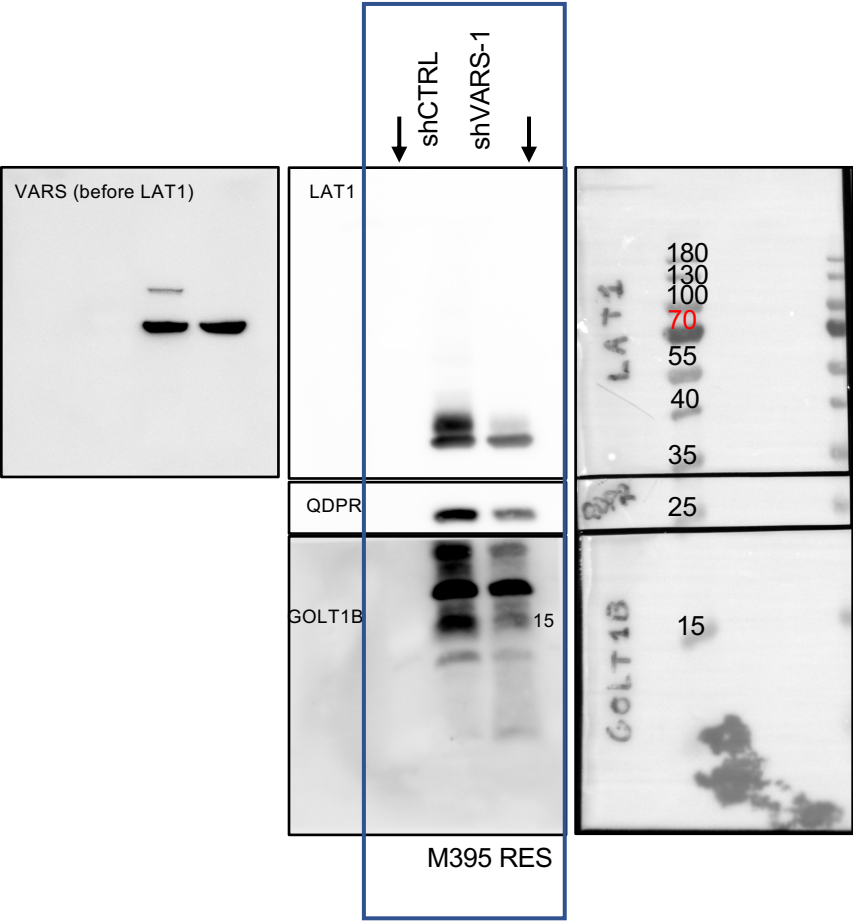

Supplement: Supplementary file 8 — Unprocessed western blots/gels. [file 41556_2024_1439_MOESM8_ESM.pdf]

Extended data figure 2d

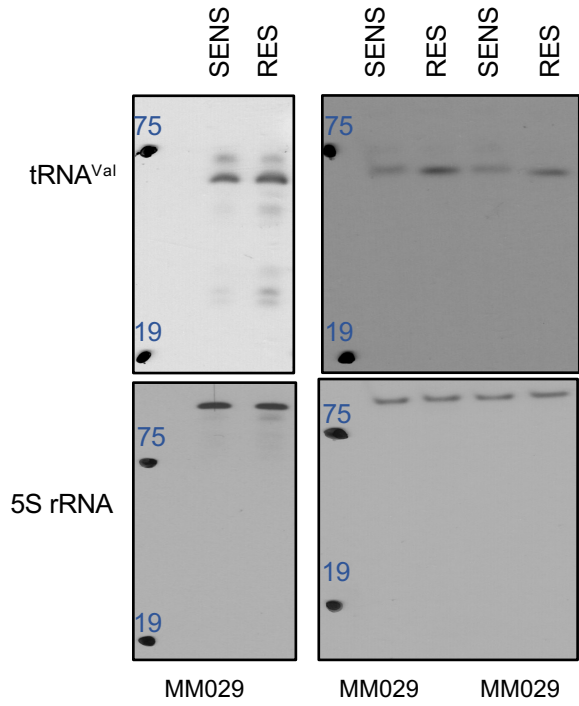

Supplement: Supplementary file 10 — Unprocessed western blots/gels. [file 41556_2024_1439_MOESM10_ESM.pdf]

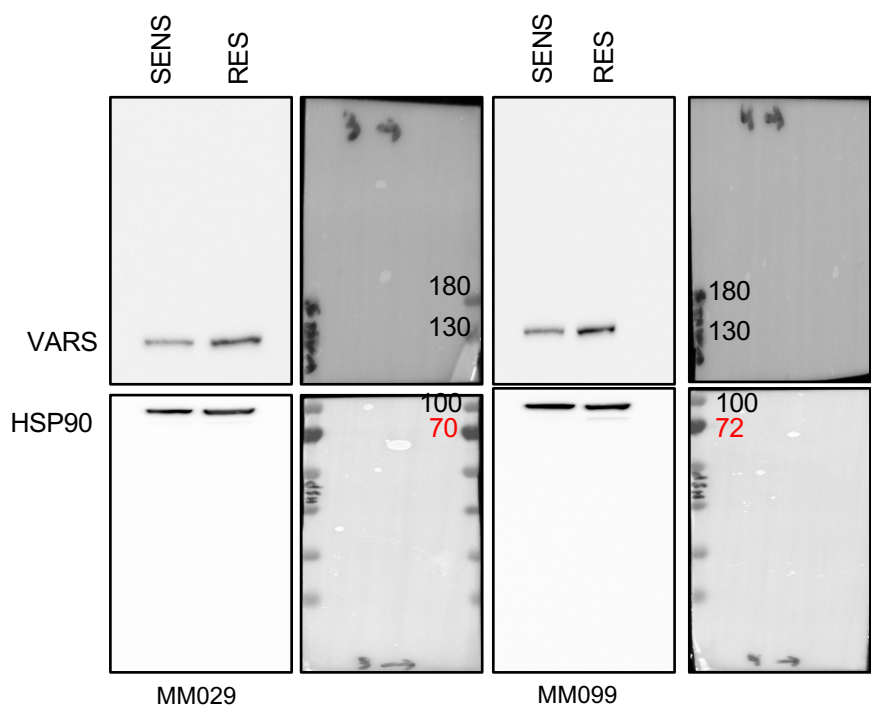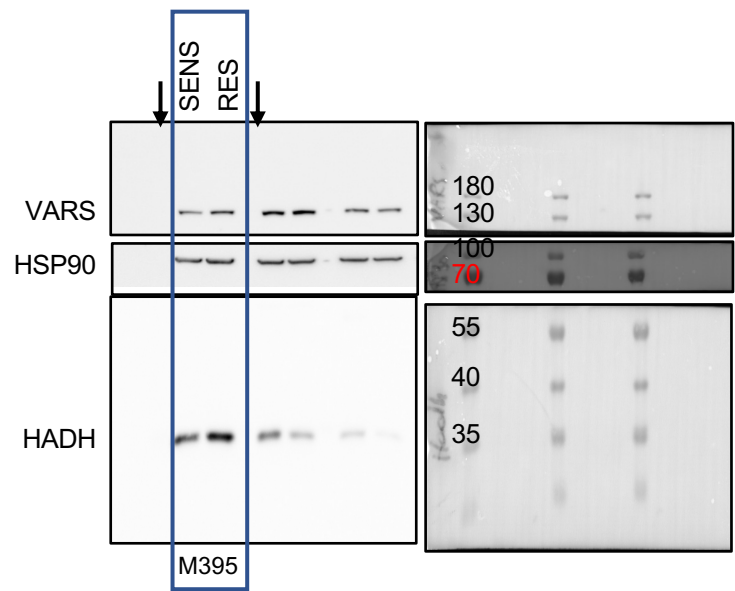

Supplement: Supplementary file 11 — Unprocessed western blots/gels. [file 41556_2024_1439_MOESM11_ESM.pdf]

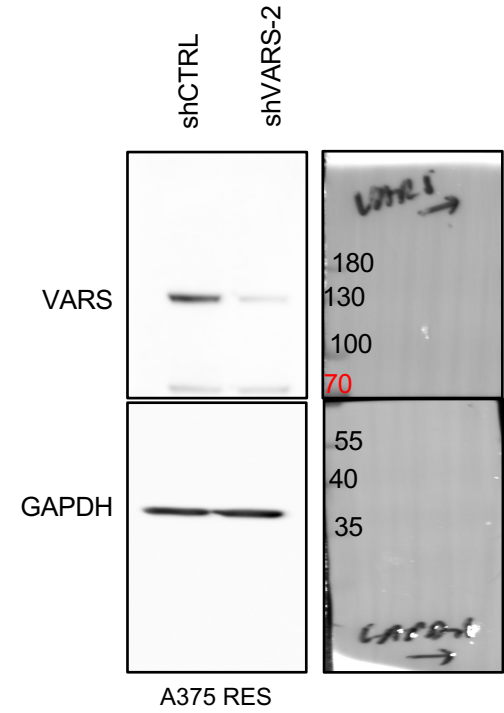

Supplement: Supplementary file 12 — Unprocessed western blots/gels. [file 41556_2024_1439_MOESM12_ESM.pdf]

Related to Extended data figure 5a

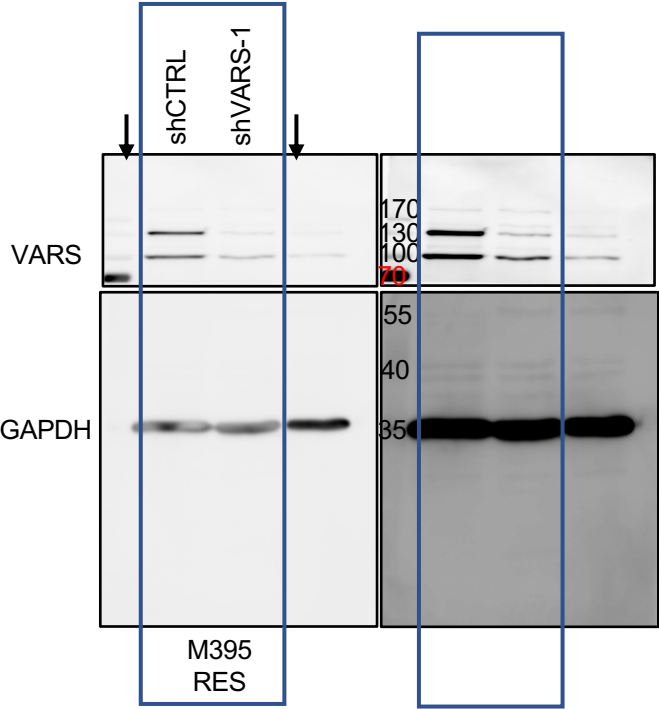

Related to Extended data figure 5b

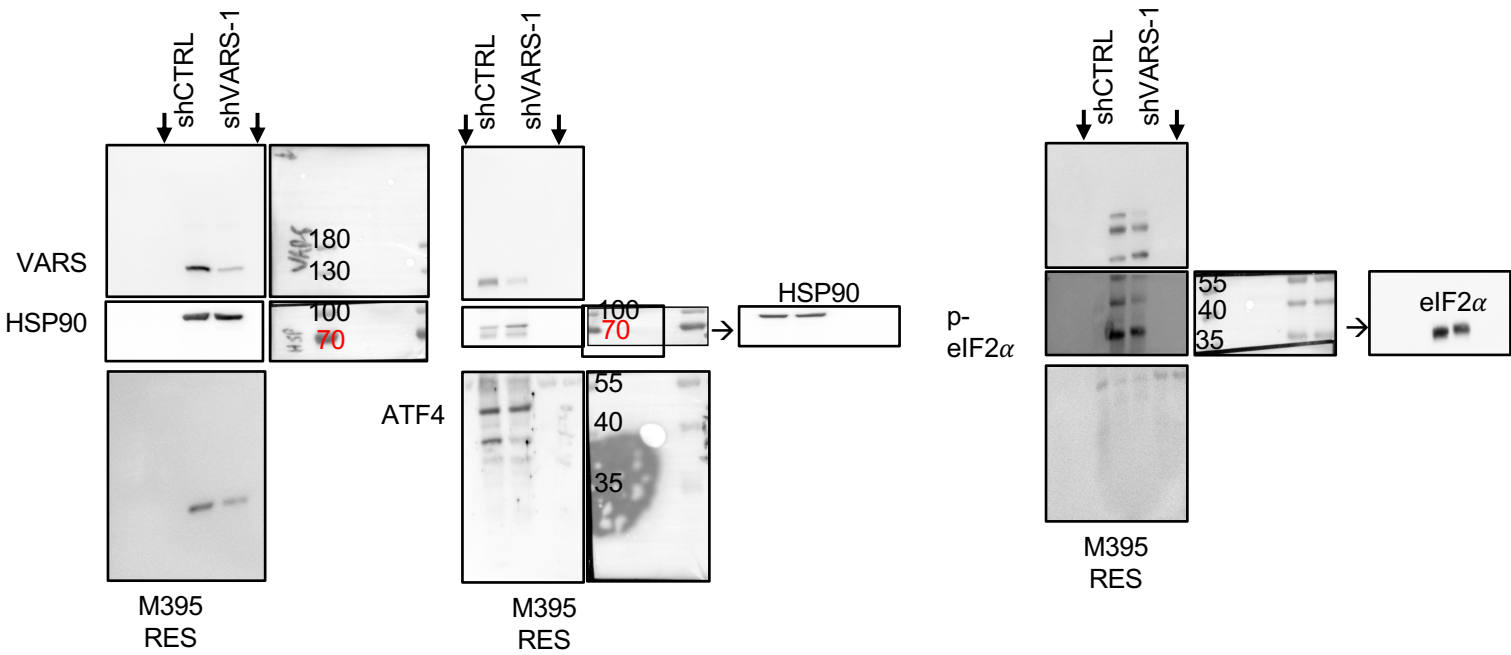

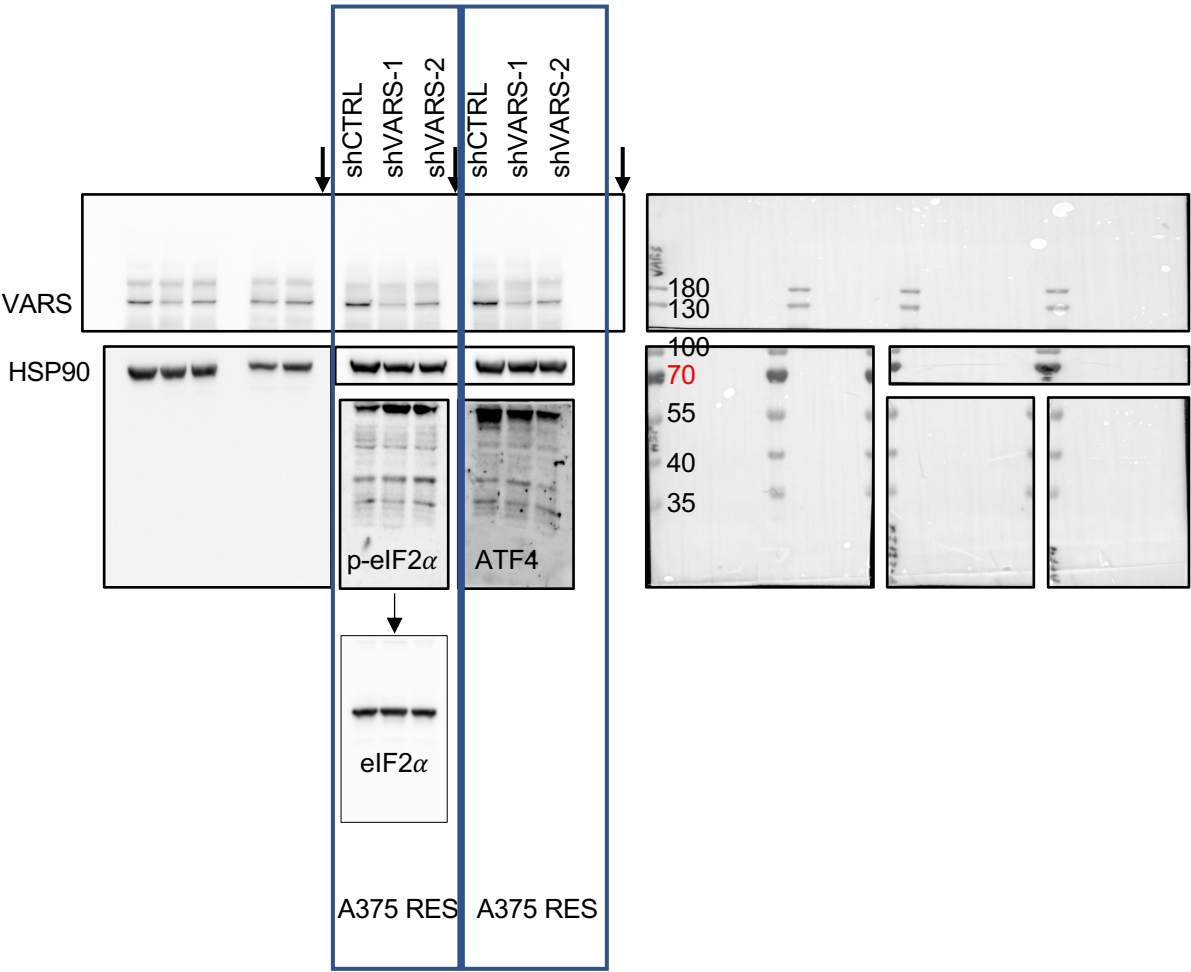

Supplement: Supplementary file 13 — Unprocessed western blots/gels. [file 41556_2024_1439_MOESM13_ESM.pdf]
