# Supplementary material for: Valine aminoacyl-tRNA synthetase promotes therapy resistance in melanoma
Source: Nat Cell Biol. 2024 Jun 7;26(7):1154–64. doi: 10.1038/s41556-024-01439-2 (PMC11252002; doi:10.1038/s41556-024-01439-2)

Related to figure 4a

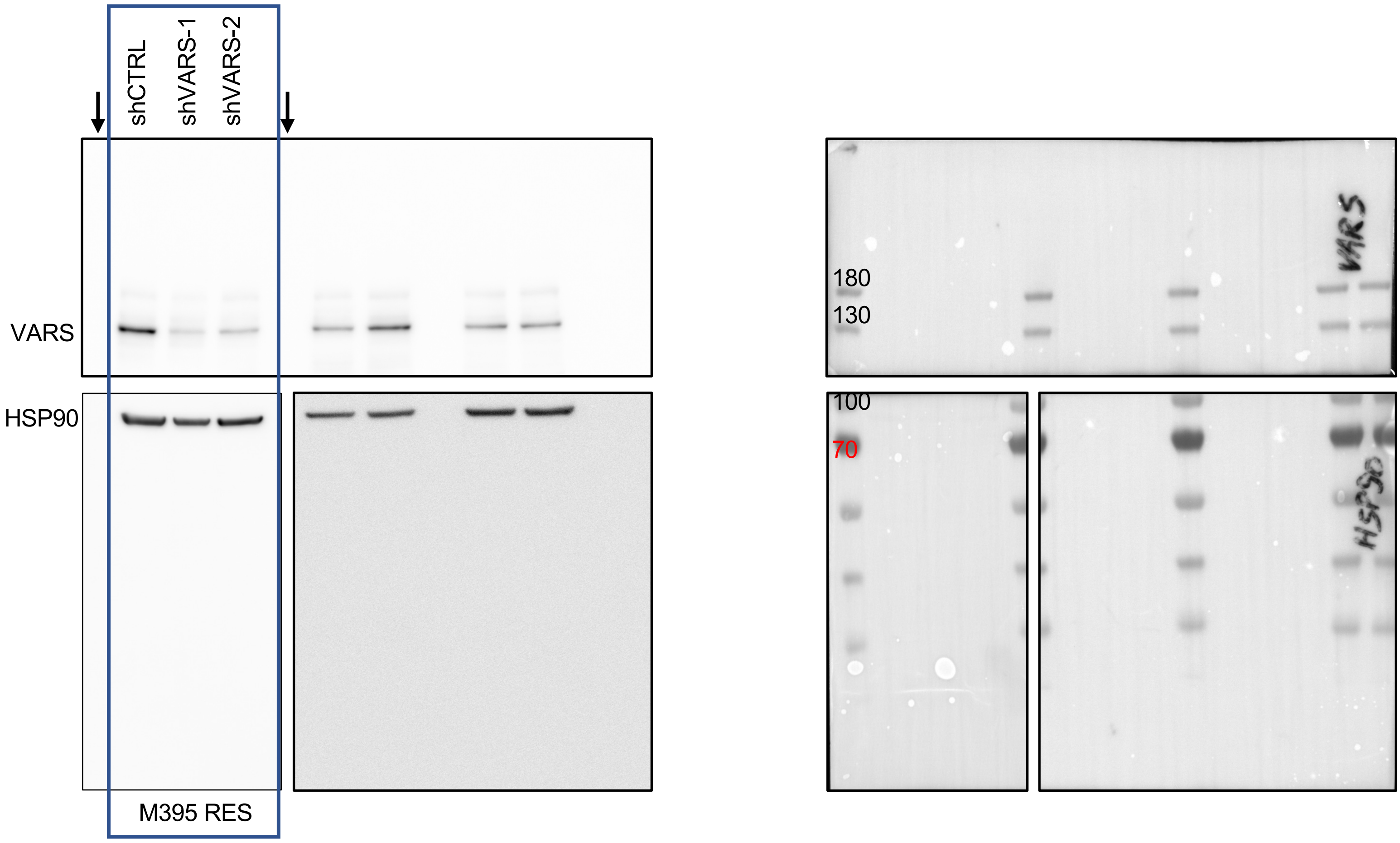

Related to figure 4b

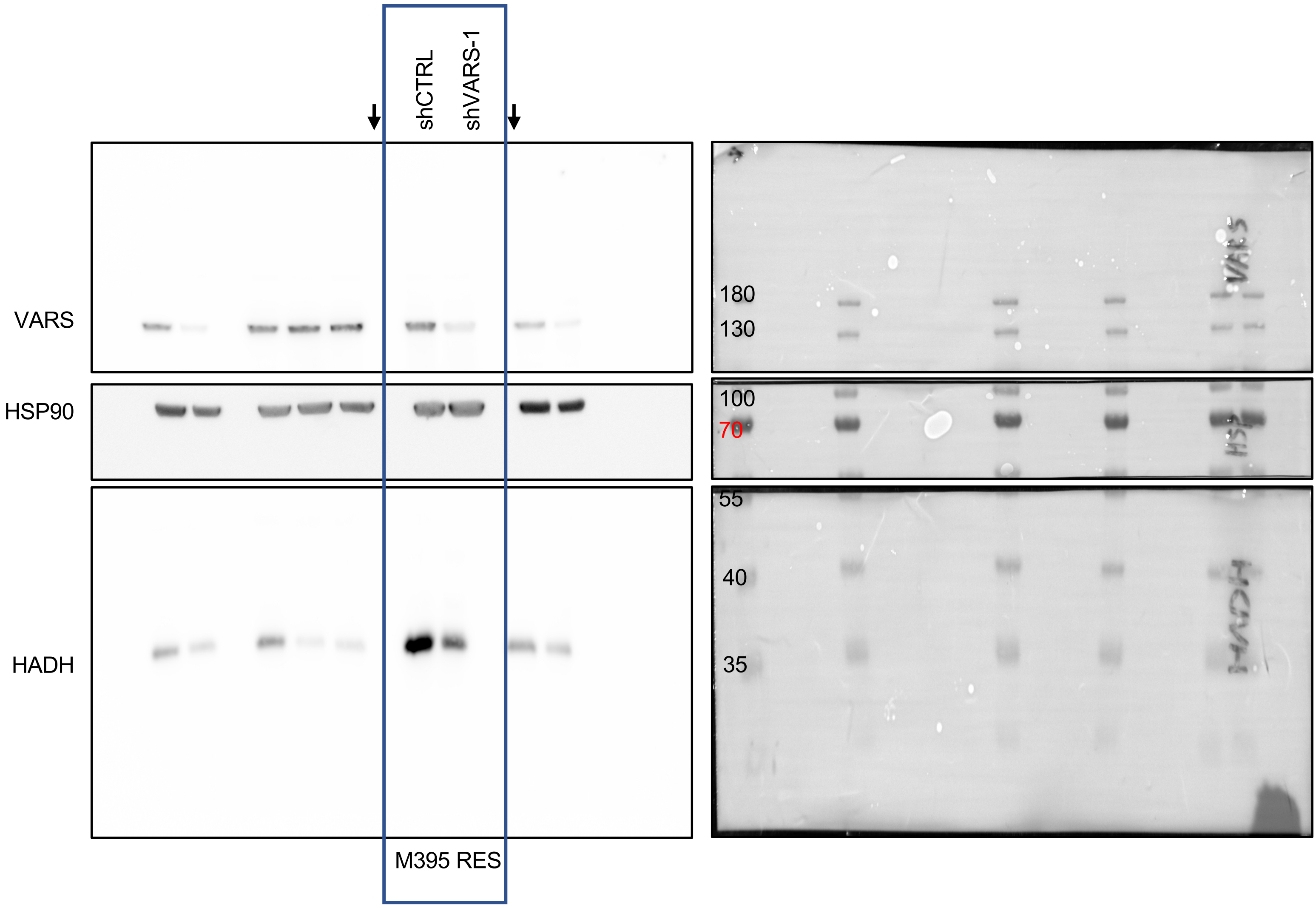

Related to figure 4c

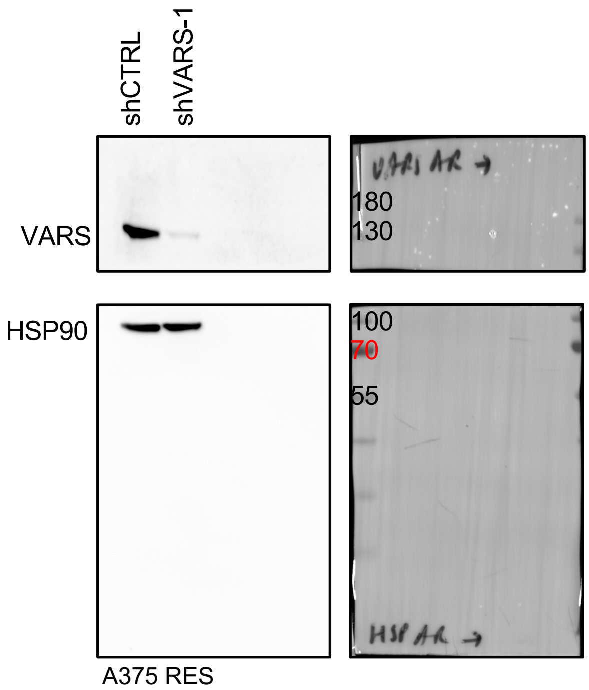

Related to figure 4d

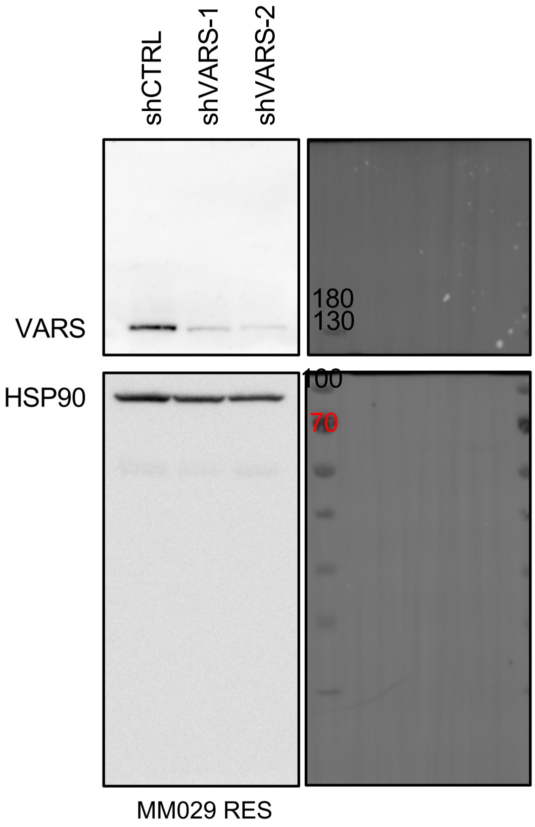

Related to figure 4d

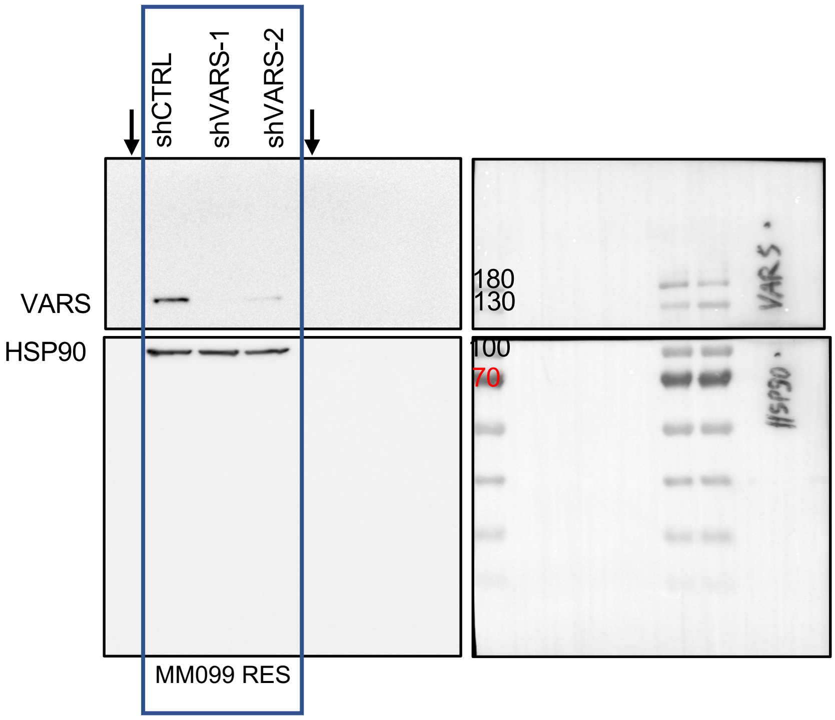

Related to figure 4d

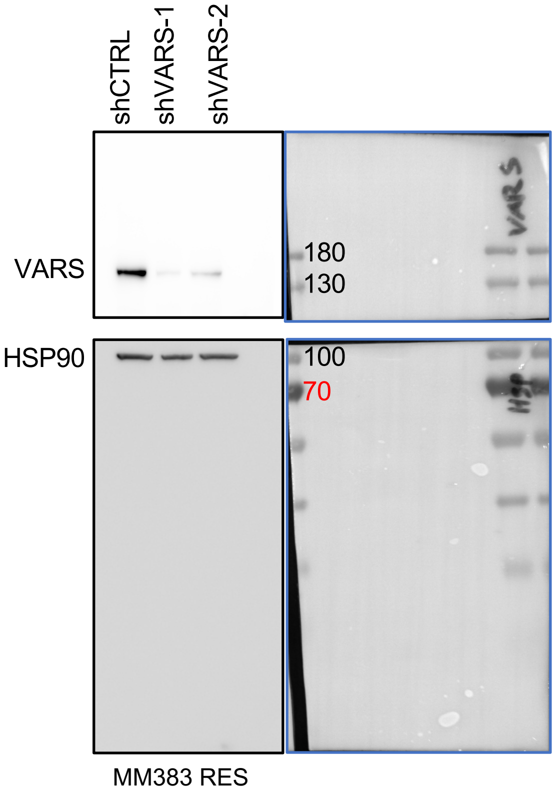

Related to figure 4f

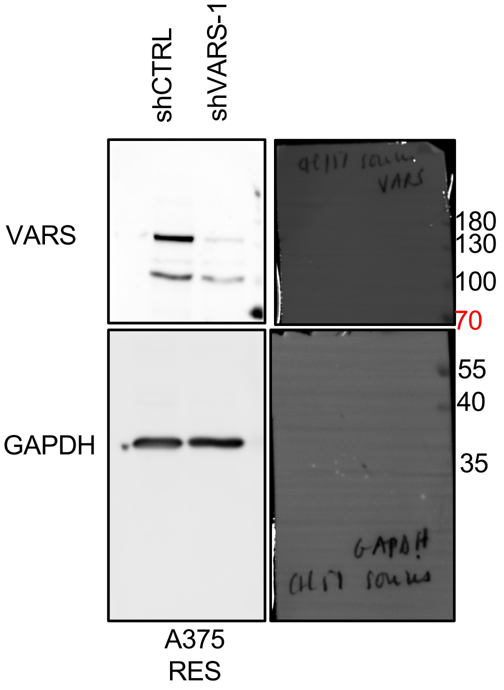

Related to figure 4e

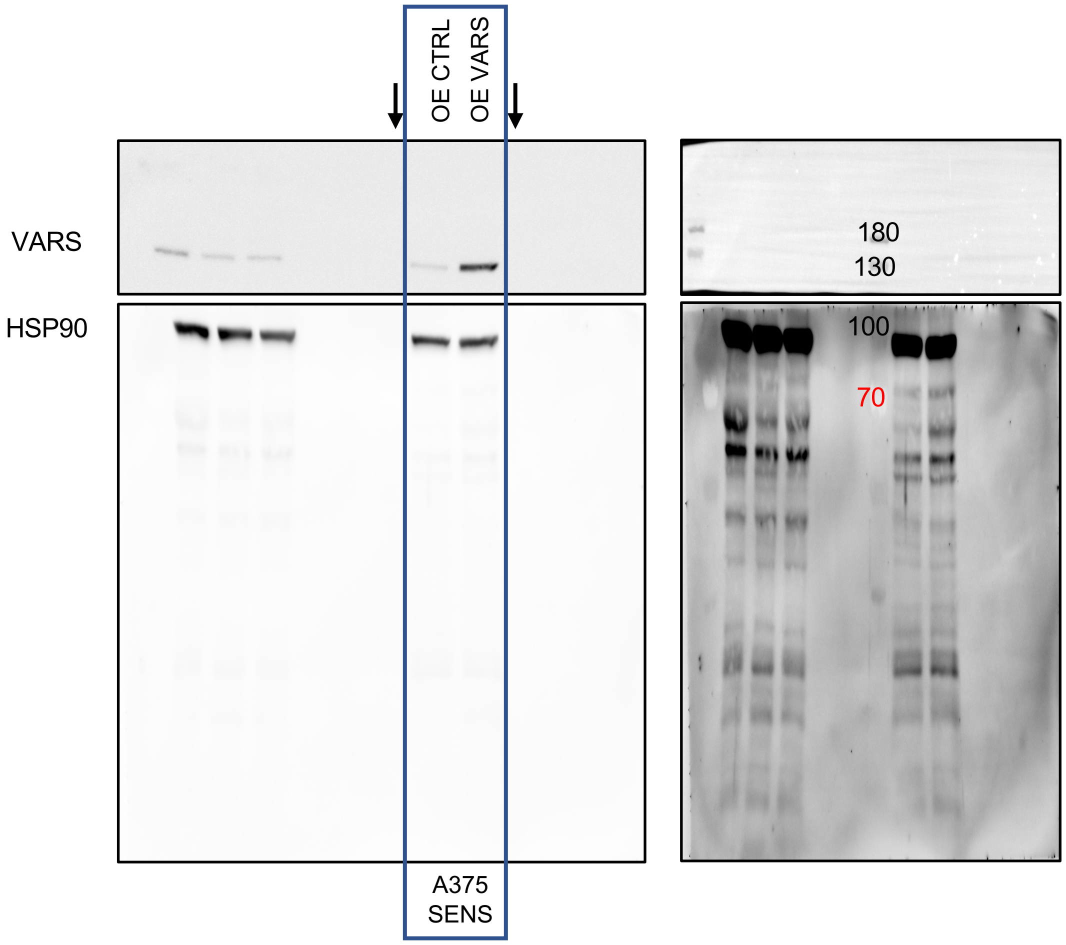

Supplement: Supplementary file 7 — Unprocessed western blots/gels. [file 41556_2024_1439_MOESM7_ESM.pdf]
