# Supplementary material for: Valine aminoacyl-tRNA synthetase promotes therapy resistance in melanoma
Source: Nat Cell Biol. 2024 Jun 7;26(7):1154–64. doi: 10.1038/s41556-024-01439-2 (PMC11252002; doi:10.1038/s41556-024-01439-2)

Related to figure 7d

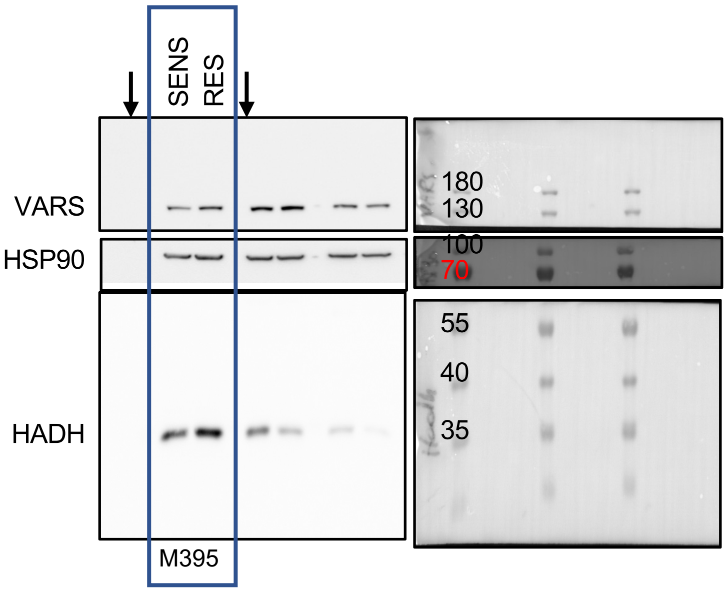

Related to figure 7e Related to figure 7f

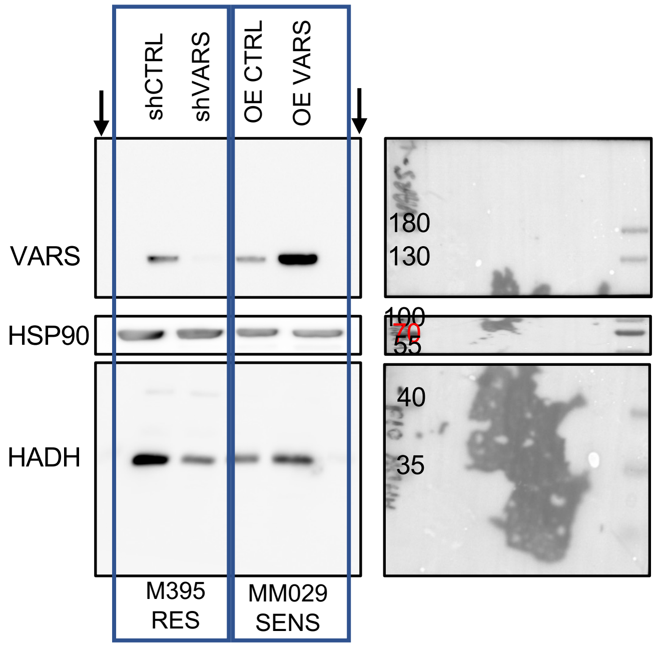

Related to figure 7f

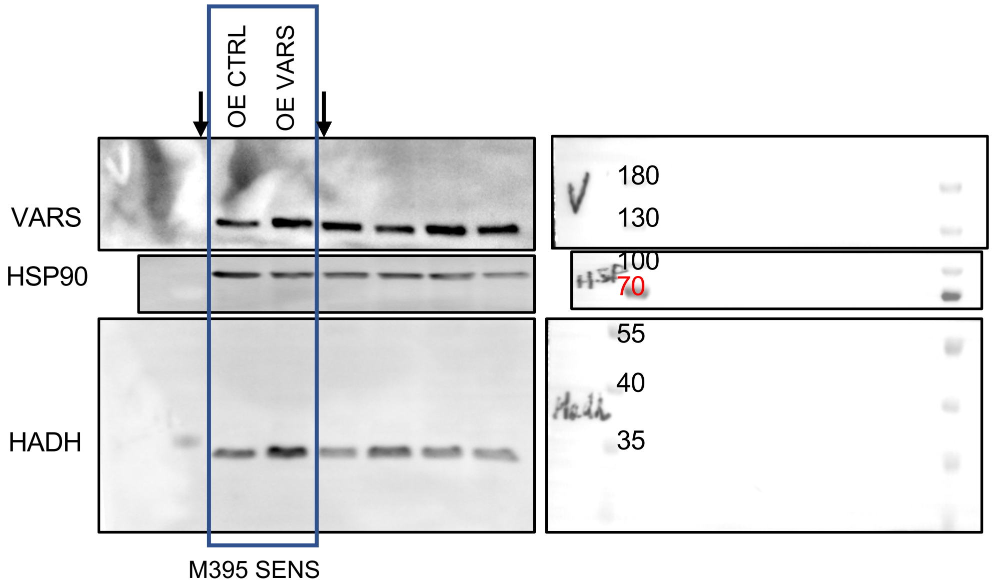

Related to figure 7e

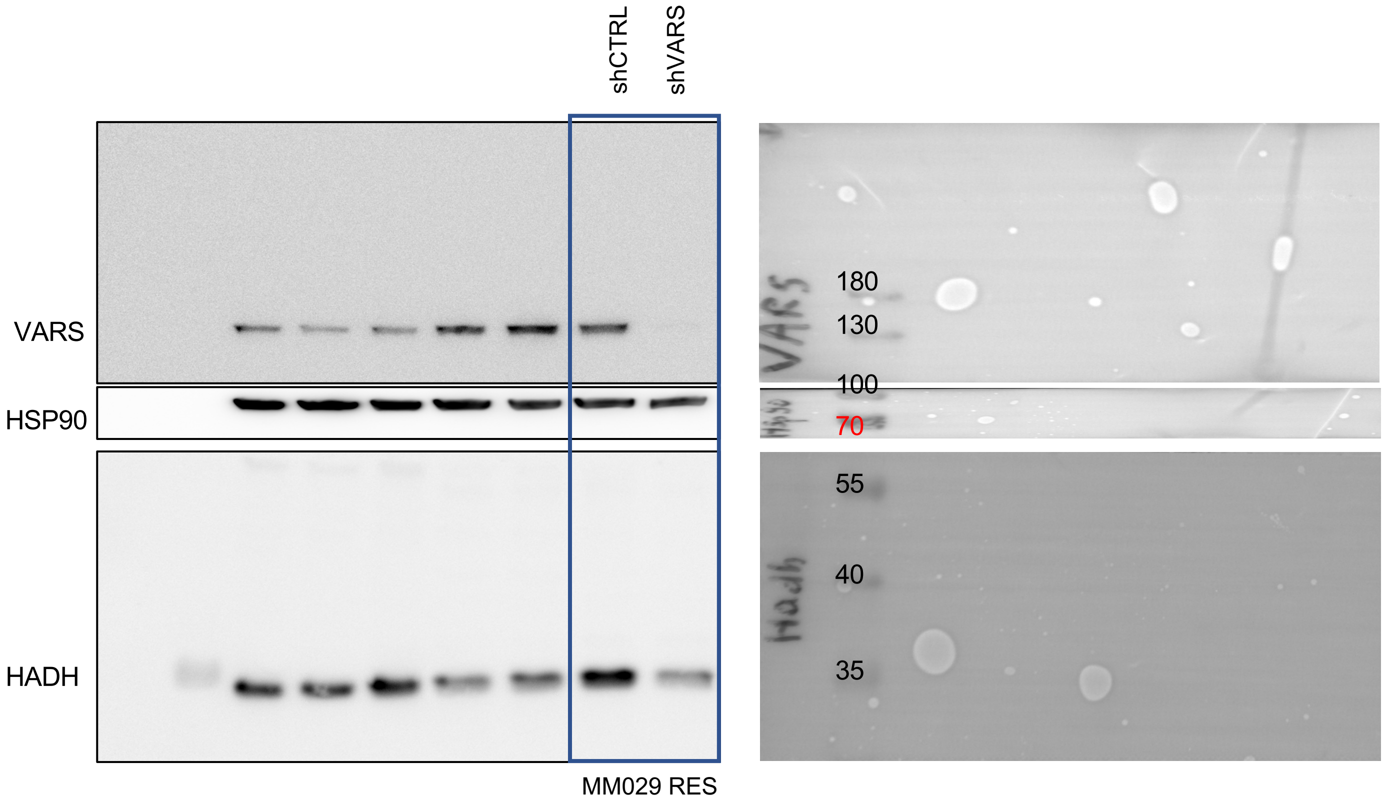

Related to figure 7f

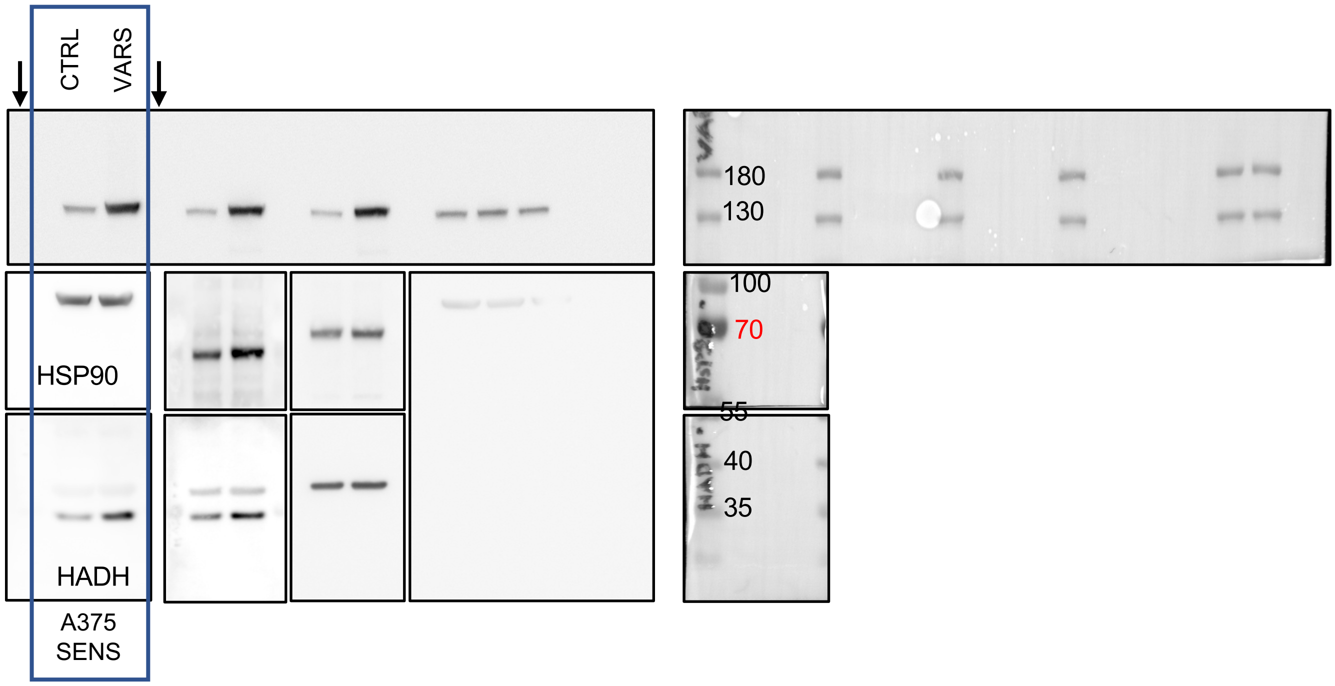

Related to figure 7e

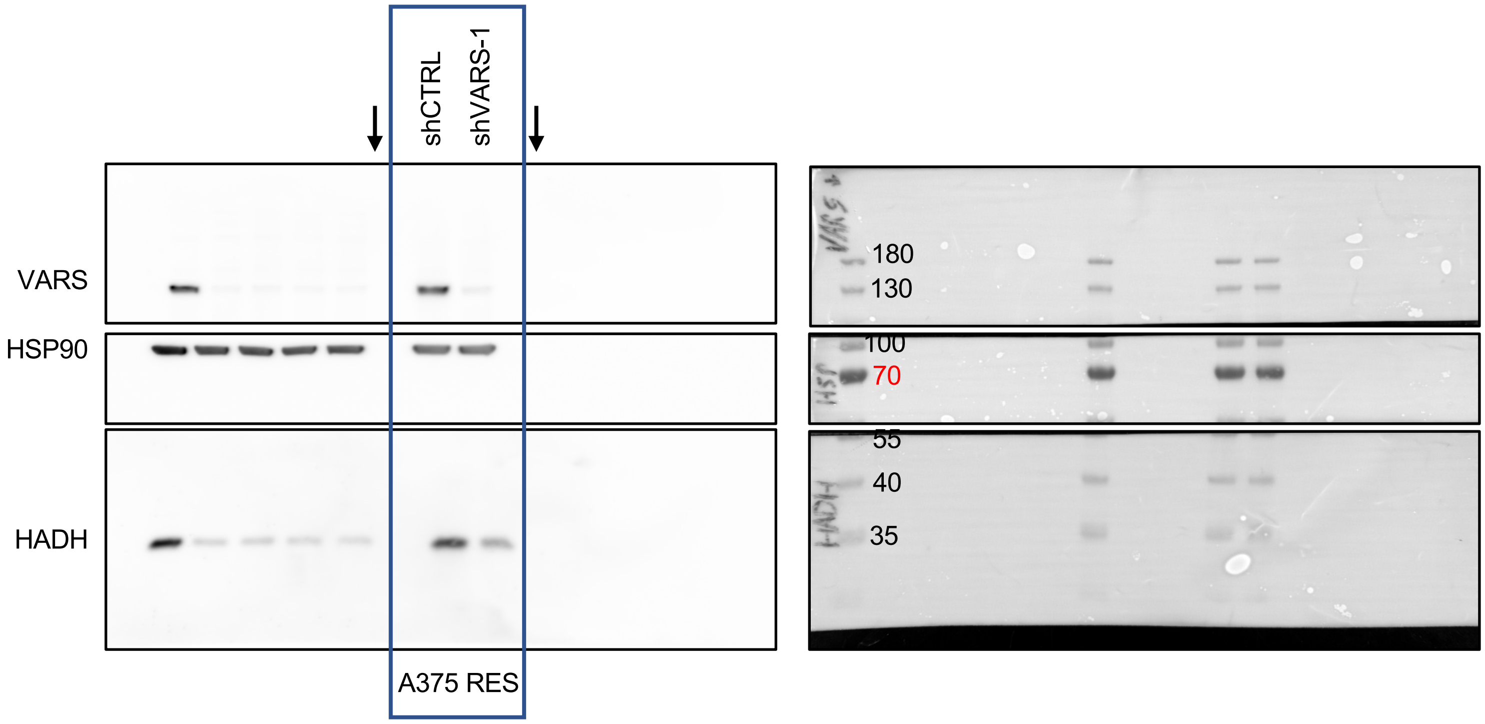

Supplement: Supplementary file 9 — Unprocessed western blots/gels. [file 41556_2024_1439_MOESM9_ESM.pdf]
