# Supplementary material for: Valine aminoacyl-tRNA synthetase promotes therapy resistance in melanoma
Source: Nat Cell Biol. 2024 Jun 7;26(7):1154–64. doi: 10.1038/s41556-024-01439-2 (PMC11252002; doi:10.1038/s41556-024-01439-2)

Related to Extended data figure 7b

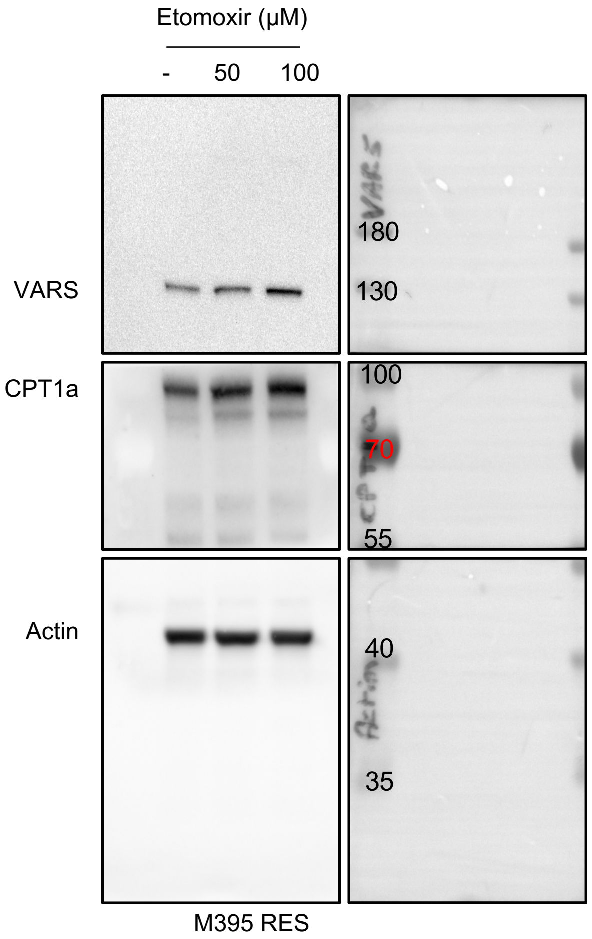

Related to Extended data figure 7d

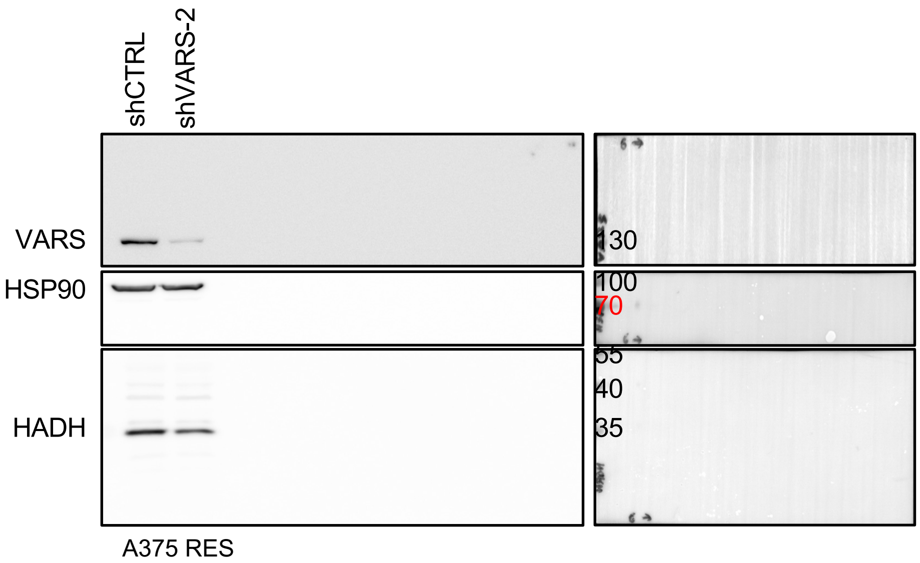

Related to Extended data figure 7d

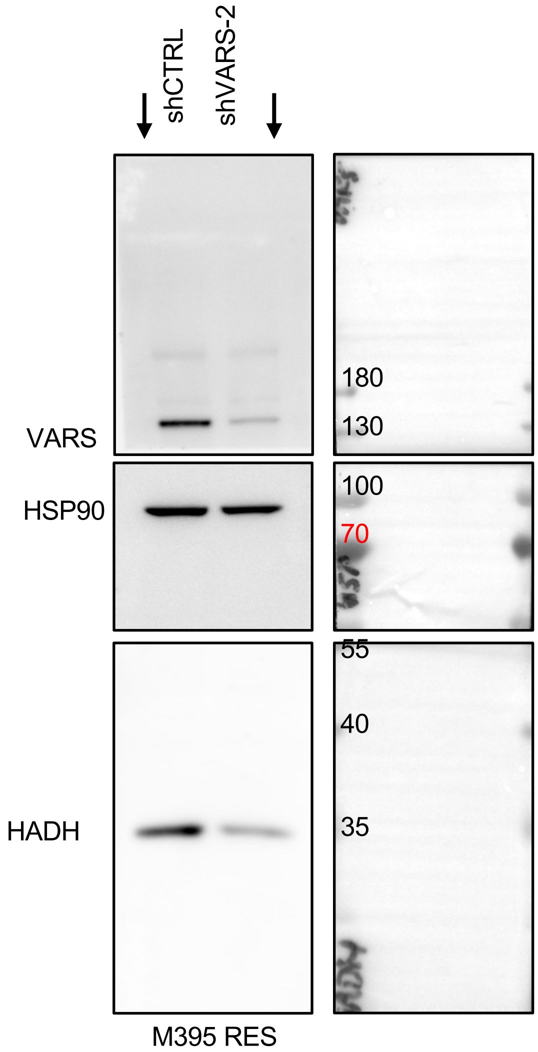

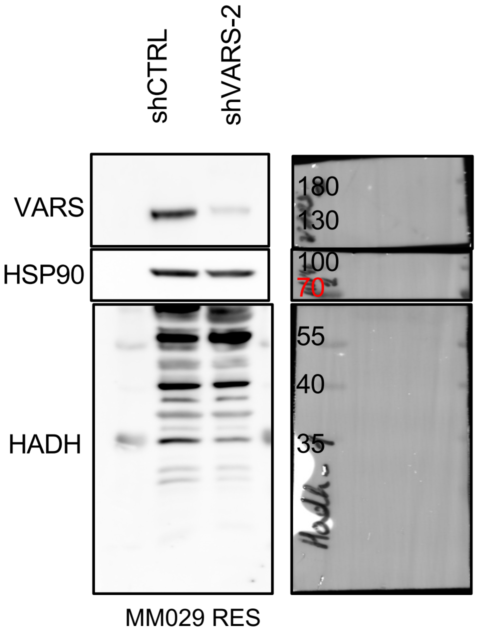

Related to
Extended data figure 7f

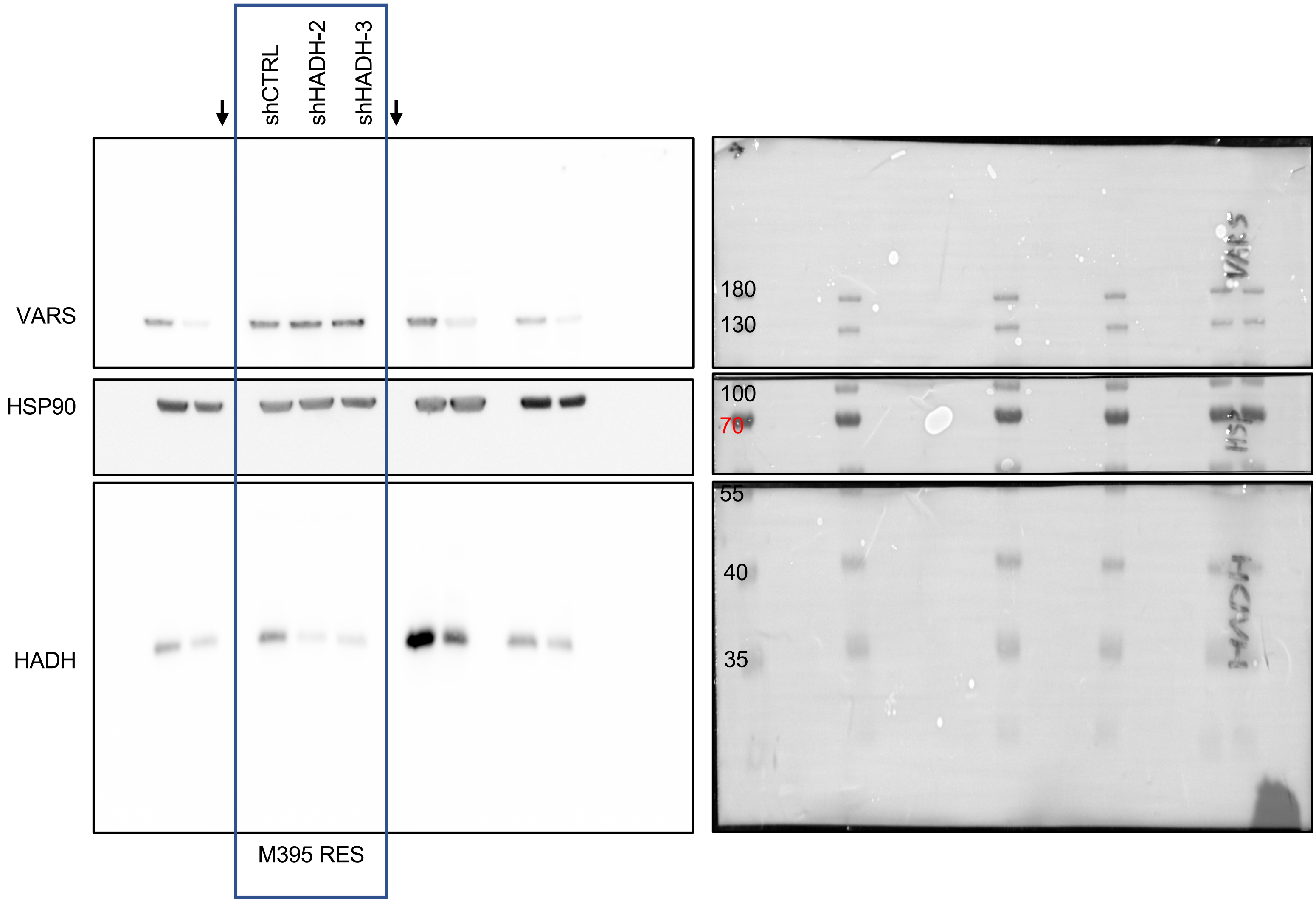

Related to
Extended data figure 7f

Related to
Extended data figure 7g

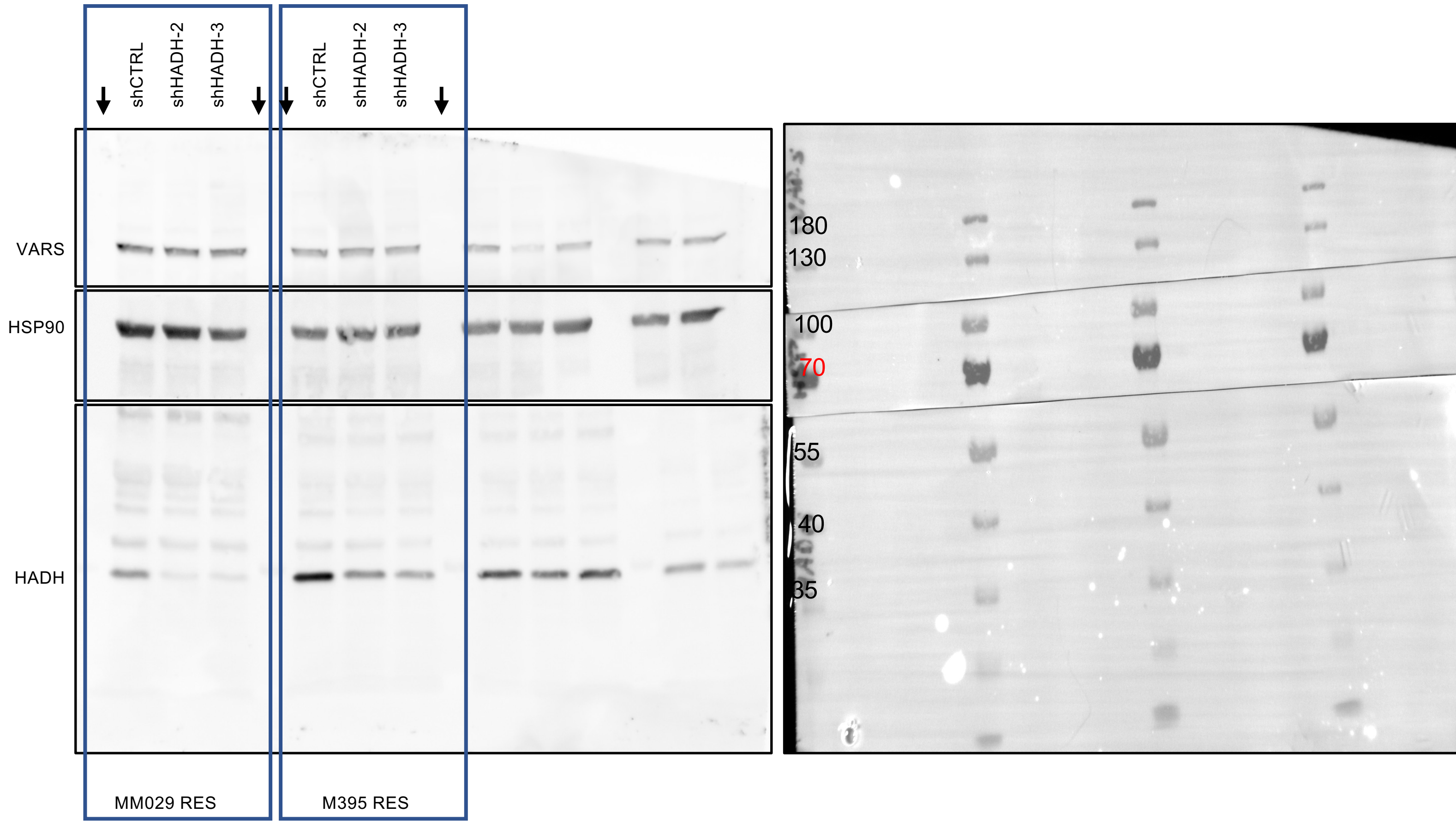

Related to Extended data figure 7h

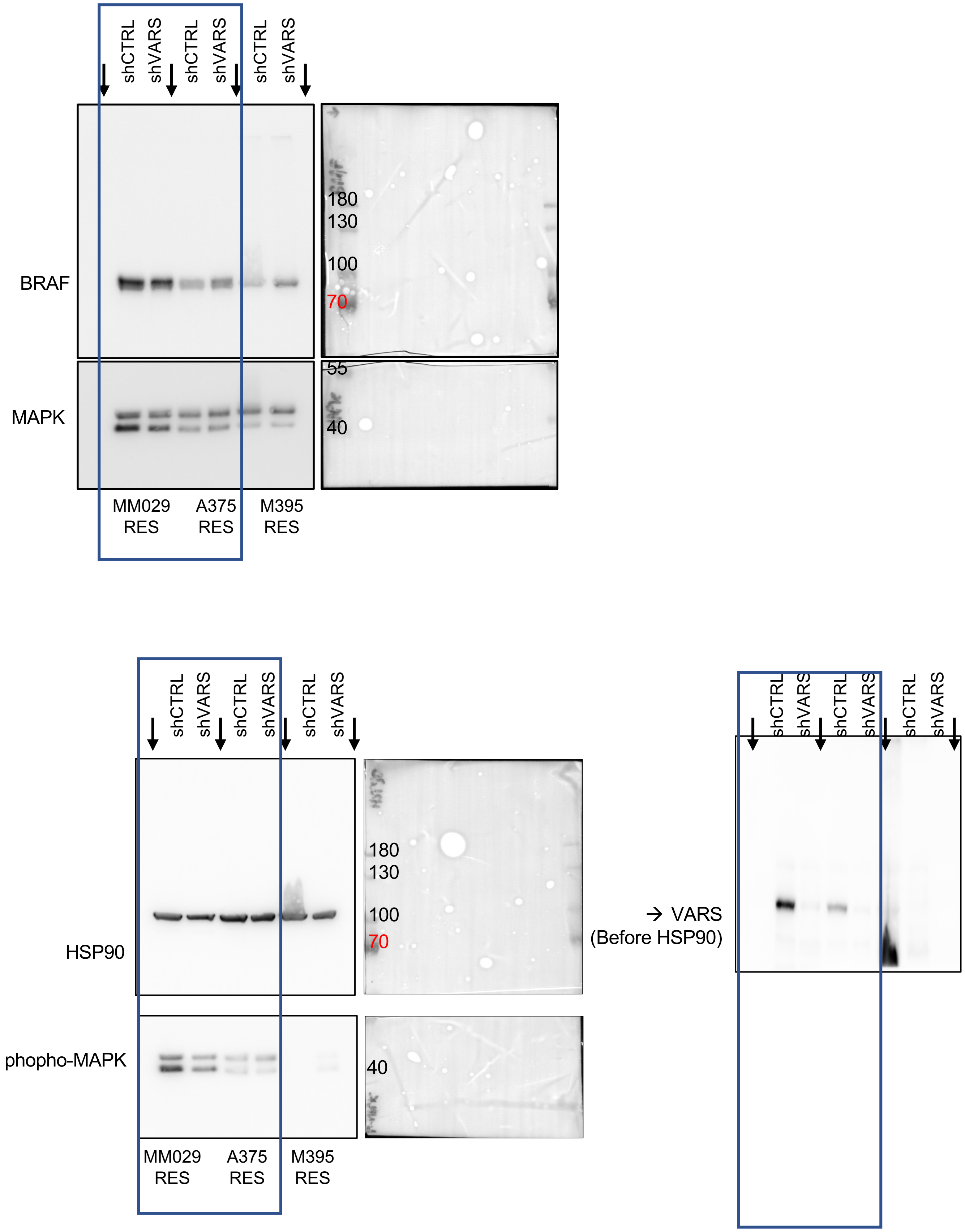

Related to Extended data figure 7h

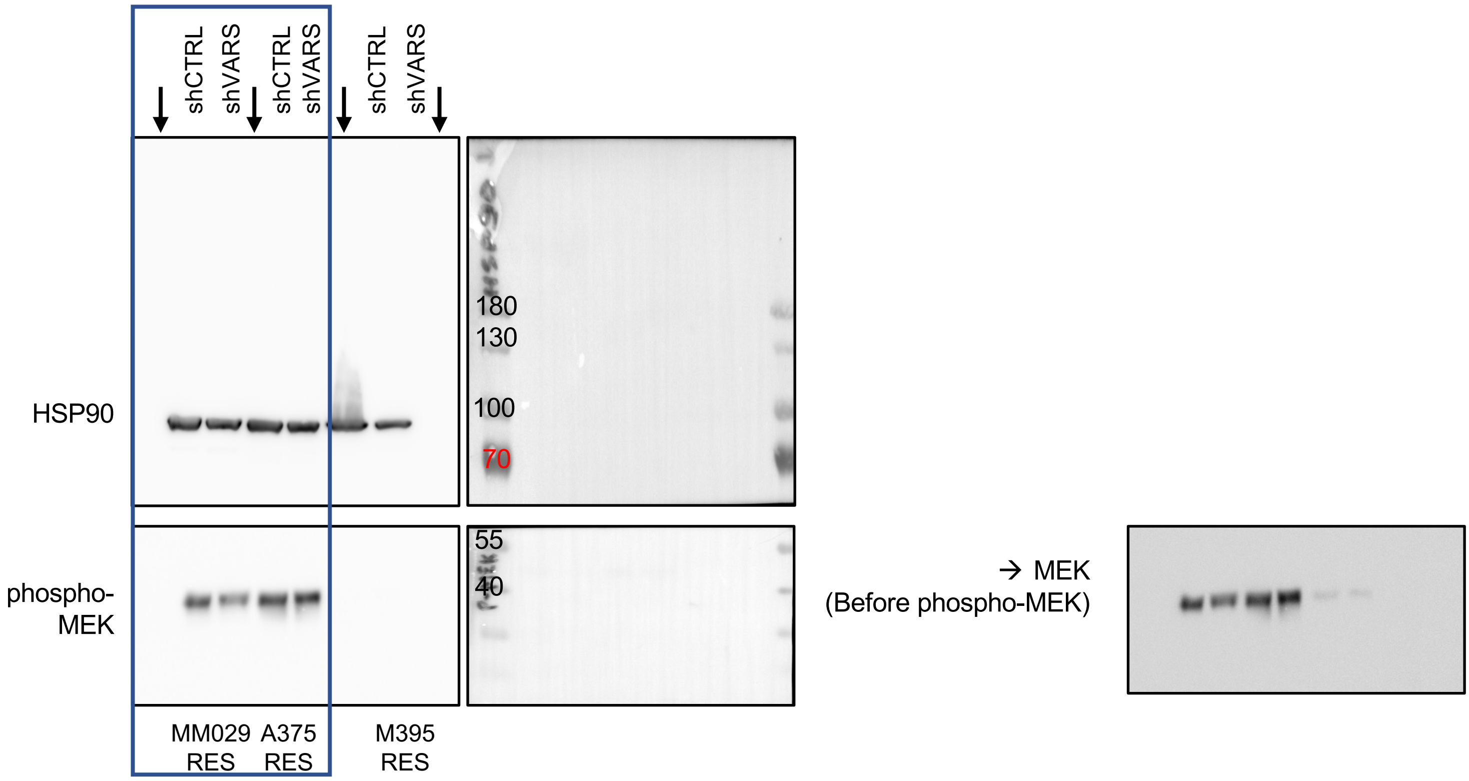

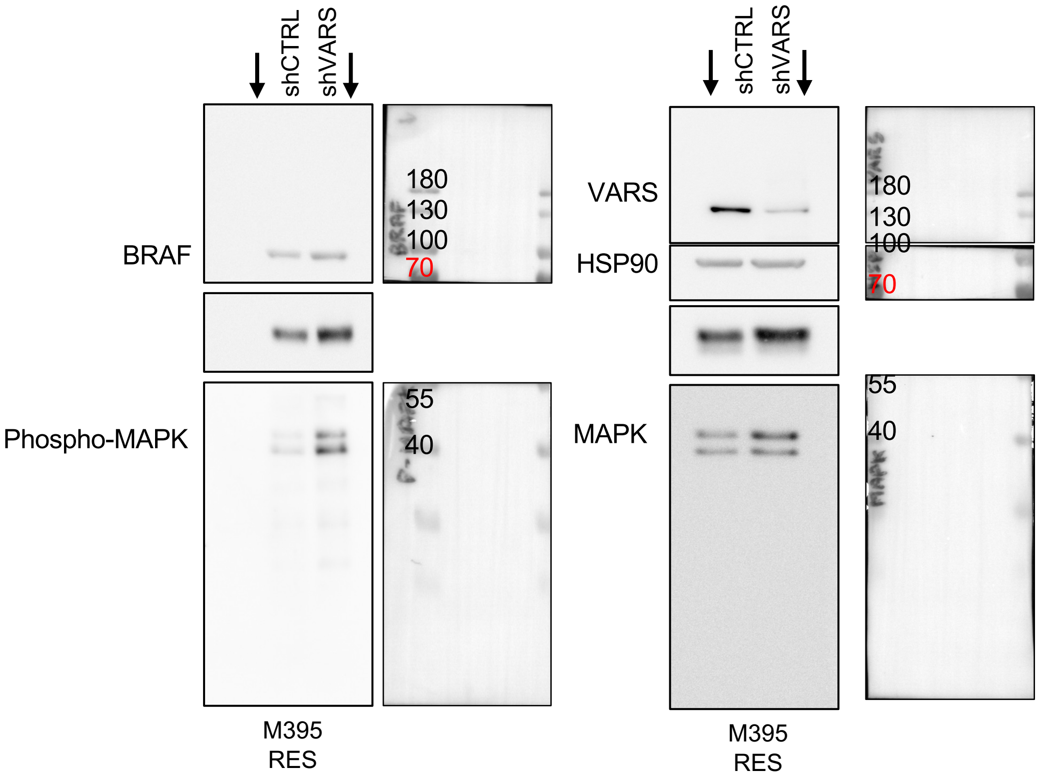

Related to Extended data figure 7h

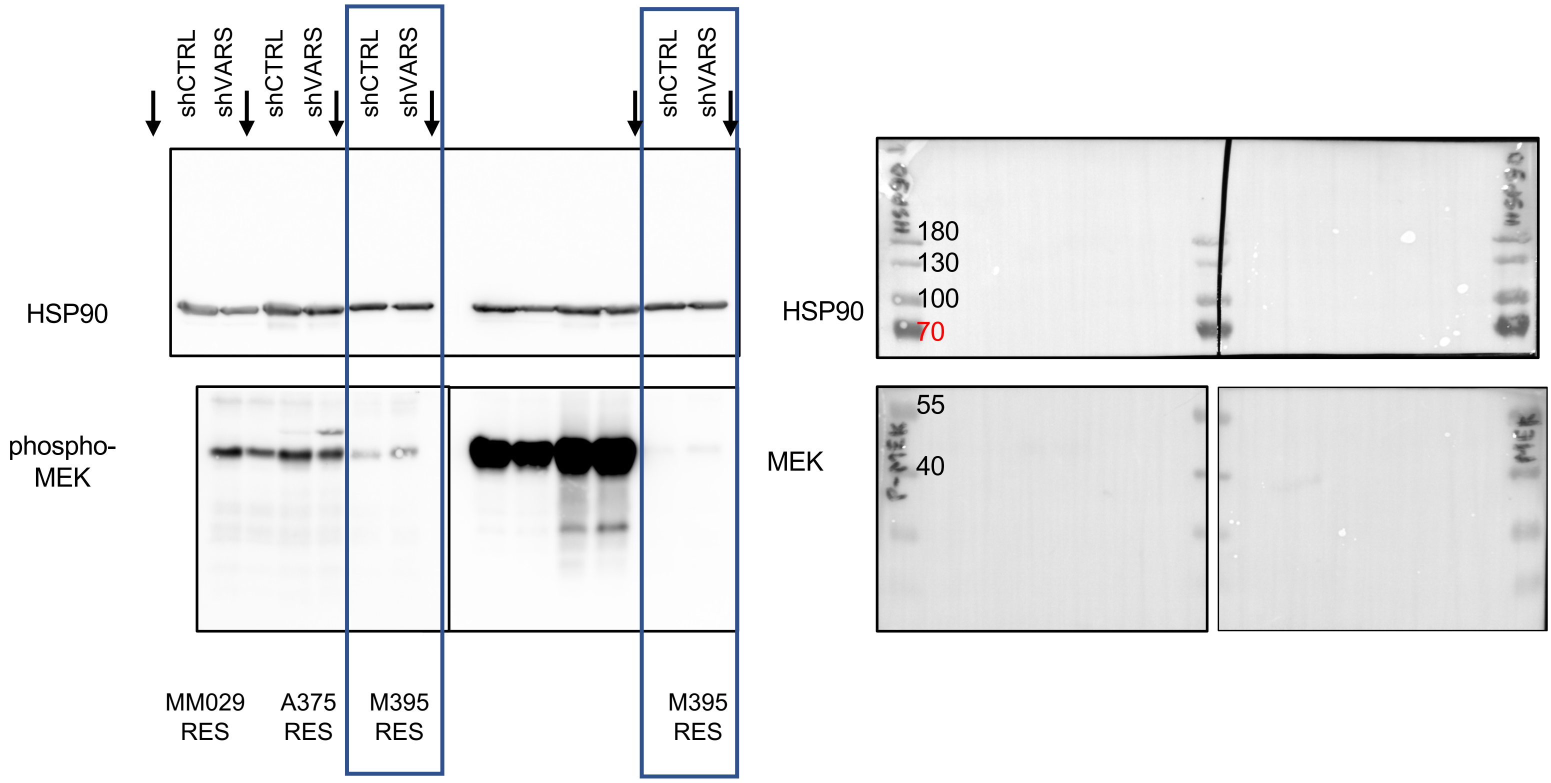

Related to Extended data figure 7i

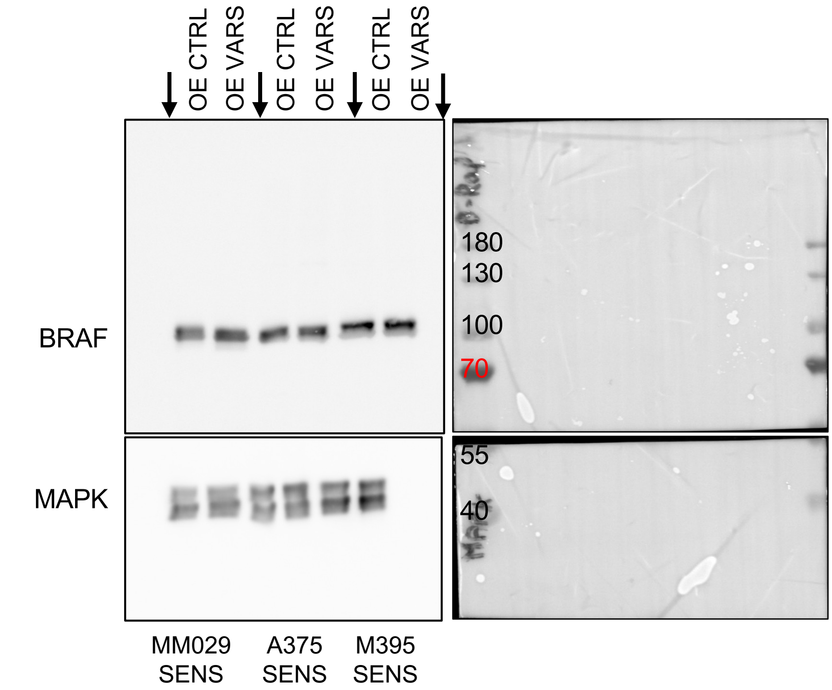

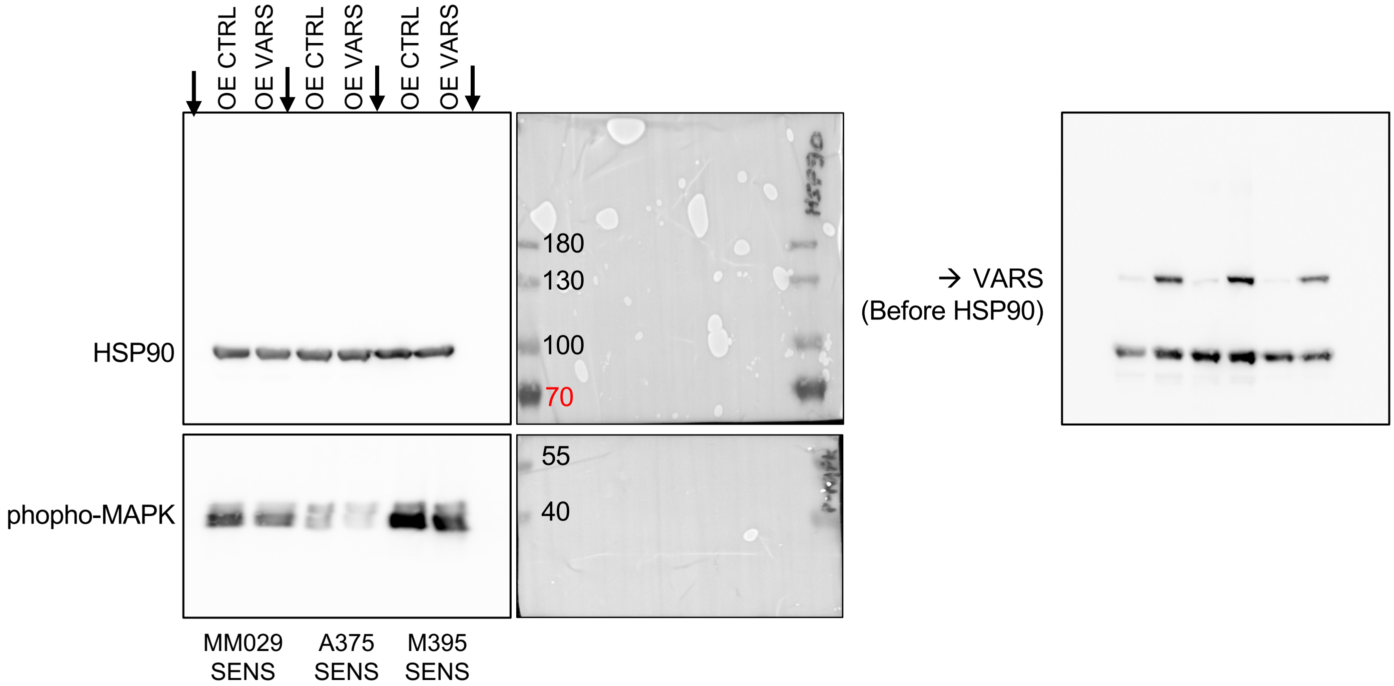

Related to Extended data figure 7i

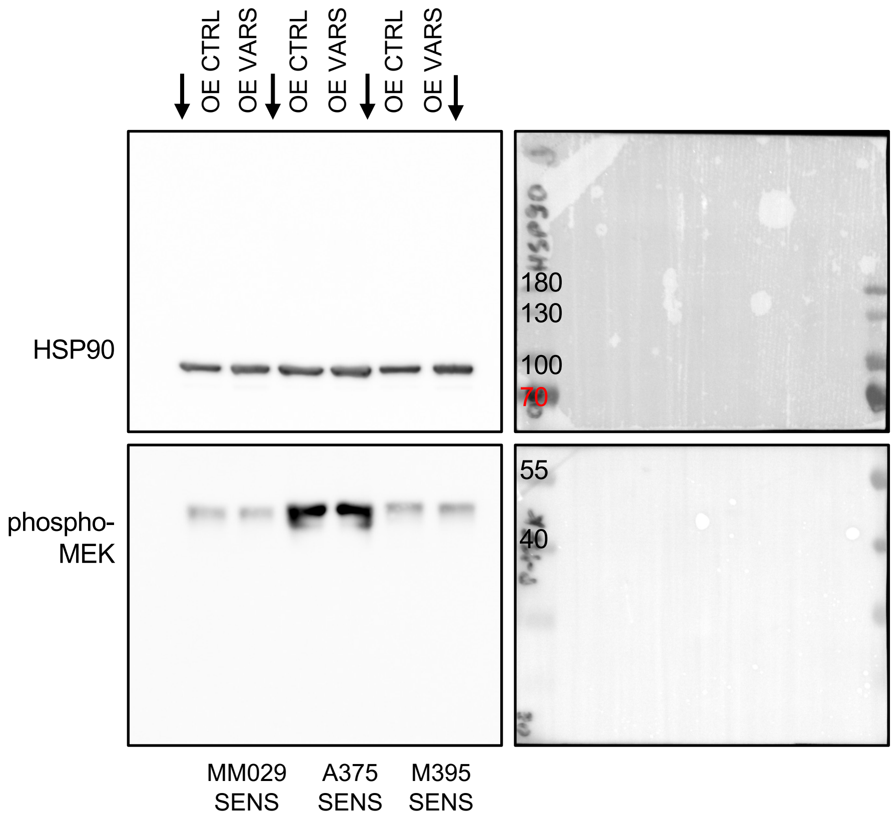

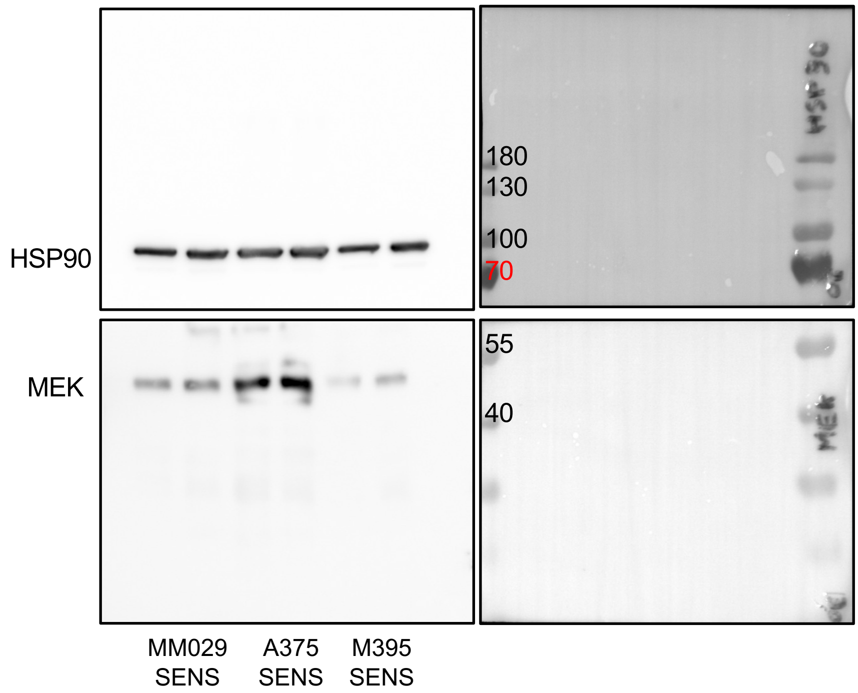

Supplement: Supplementary file 14 — Unprocessed western blots/gels. [file 41556_2024_1439_MOESM14_ESM.pdf]
